# Nanostructured Carbon-Nitrogen-Sulfur-Nickel Networks Derived From Polyaniline as Bifunctional Catalysts for Water Splitting

**DOI:** 10.3389/fchem.2020.00385

**Published:** 2020-05-20

**Authors:** Razik Djara, Yaovi Holade, Abdelhafid Merzouki, Marie-Agnès Lacour, Nathalie Masquelez, Valerie Flaud, Didier Cot, Bertrand Rebiere, Arie van der Lee, Julien Cambedouzou, Patrice Huguet, Sophie Tingry, David Cornu

**Affiliations:** ^1^Laboratoire de Physico-Chimie des Hauts Polymères (LPCHP), Université Ferhat Abbas, Sétif, Algeria; ^2^Institut Européen des Membranes, IEM UMR 5635, Univ Montpellier, ENSCM, CNRS, Montpellier, France; ^3^ENSCM, ChemLab, Montpellier, France; ^4^Institut Charles Gerhardt, ICGM UMR 5253, Univ Montpellier, ENSCM, CNRS, Montpellier, France

**Keywords:** polyaniline, hydrogen evolution reaction, oxygen evolution reaction, water splitting, electrocatalysis, noble metal-free, nickel, nitrogen doping

## Abstract

The development of reliable production routes for sustainable hydrogen (H_2_), which is an essential feedstock for industrial processes and energy carrier for fuel cells, is needed. It appears to be an unavoidable alternative to significantly reduce the dependence on conventional energy sources based on fossil fuels without increasing the atmospheric CO_2_ levels. Among the different power-to-X scenarios to access high purity H_2_, the electrochemical approach based on electrolysis looks to be a promising sustainable solution at both the small and large industrial scales. However, the practical realization of this important opportunity faces several challenges, including the efficient design of cost-effective catalytic materials to be used as a cathode with improved intrinsic and durable activity. In this contribution, we report the design and development of efficient nanostructured catalysts for the electrocatalytic hydrogen evolution reaction (HER) and oxygen evolution reaction (OER) in aqueous media, whereby noble metal-free elements are embedded in a matrix of a conducting polymer, polyaniline (PANI). To increase the electrical conductivity and further the electrocatalytic ability toward HER of the chemically polymerized PANI in the presence of nickel (II) salt (nitrate), the PANI-based materials have first been stabilized at a mild temperature of 250–350°C in air and then carbonized at 800–1,000°C under nitrogen gas to convert the chemical species into nitrogen, sulfur, nickel, and carbon nanostructured networks (CNNs). Different physicochemical (TGA-DSC, Raman spectroscopy, XRD, SEM, EDX, ICP, CHNS, BET, and XPS) and electrochemical (voltammetry and electrochemical impedance spectrometry) methods have been integrated to characterize the as-synthesized CNNs materials and interrogate the relationship of material-to-performance. It has been found that those synthesis conditions allow for the substantial increase of the electrocatalytic performance toward HER and OER in alkaline media in terms of the onset potential and charge transfer resistance and overpotential at the specific activity of 10 milliamps per square centimeter, thus ranking the present materials among the most efficient noble metal-free catalysts and making them possible candidates for integration in practical low-energy consumption alkaline electrolyzers.

## Introduction

Molecular hydrogen (H_2_) plays a pivotal role in various industrial processes as a feedstock and an energy carrier involved in energy conversion and storage scenarios through electrolysis and fuel cells (Montoya et al., 2017; Stamenkovic et al., 2017; Hacker and Mitsushima, 2018; Kumar and Himabindu, 2019; Staffell et al., 2019). The search for “natural” sources of harvesting H_2_ has received renewed interest, but to date, there are no facilities providing a substantial supply (Welhan and Craig, 1979; Guélard et al., 2017). Hence, to cover the continued increase in end-uses, H_2_ is synthetically/artificially produced by chemical methods. These methods mean that more than 96% of H_2_ comes from fossil fuels in a variety of processes that also generate CO_2_ (Armaroli and Balzani, 2011; Zou and Zhang, 2015; Mitsushima and Hacker, 2018; Voitic et al., 2018). The resulting H_2_ must be cleaned of carbonaceous impurities before use, which adds complexity and additional costs. These strategies do not meet the requirements of decarbonized and sustainable development as well as the high-purity H_2_ demands. Therefore, one of the most effective alternative routes for the production of high-purity H_2_ is water splitting (2H_2_O → 2H_2_ + O_2_), a technology in which the electrical energy required can come from many renewable sources (Seh et al., 2017).

Noble metals, and in particular Pt, Ir, and Ru-based nanostructured materials, have been considered as ideal electrocatalysts for water electrolysis by reducing the overpotential during the electrocatalytic hydrogen evolution reaction (HER, 2H_2_O + 2e^−^ → H_2_ + 2HO^−^) and oxygen evolution reaction (OER, 4HO^−^ → O_2_ + 2H_2_O + 4e^−^). But the high cost and low earth abundance of those materials have limited their use in large-scale H_2_ production technologies (Vesborg and Jaramillo, 2012; Seh et al., 2017; Stamenkovic et al., 2017). To substantially decrease the cell voltage of water electrolysis that generates a consumed electrical energy of about 53 kWh ${\text{kg}}_{\text{H}2}^{-1}$, organic electroreforming has been proposed as an alternative to OER, which would save nearly 50% of the energy used (Hasa et al., 2018; Caravaca et al., 2019). For the cathode reaction, the only solution is to develop cost-effective materials to substitute those precious metals in electrocatalysts. From this perspective, conducting polymers combined with low loading of metallic species are good candidates for high-performance electrocatalysts due to their interesting structural and physical properties (Ghosh et al., 2015, 2016, 2017; Wang et al., 2016a; Ramohlola et al., 2018). Among those polymers, polyaniline (PANI) has received tremendous attention in recent years in (bio)electrochemistry to be directly used as an electrode or indirectly as a supporting material (Wu et al., 2011; Silva et al., 2013; Boeva and Sergeyev, 2014; Xu and Minteer, 2014; Wang et al., 2016a; Cao et al., 2017; Torres et al., 2017; Feng et al., 2018; Masibi et al., 2018; Ramohlola et al., 2018; Shendkar et al., 2018; Bai et al., 2019). Torres and co-workers have shown that upon the introduction of Ni in a PANI-based electrode, the exchange current density (j_0_) increases from 0.35 to 3.47 μA cm^−2^, which indicates that the ability of electron transfer (driving force in electrocatalysis) is about one magnitude higher (Torres et al., 2017). Similar improvement of the electrocatalytic activity toward HER has been recently reported following the introduction of precursors of Fe, Co, Mn, and Ni (Feng et al., 2018). Among those metals, the performance trend is Co > Ni > Fe, Mn. The criteria of abundance and cost already exclude the Co, which is widely used in batteries. Therefore, taking into account the electrocatalytic performance, the most considered option is nickel. It should also be noted that, traditionally, bulk nickel has been the most widely used base material in liquid alkaline electrolyte technologies. Those facts justify the interest in this material in the context of the nanostructured electrocatalytic materials development for the compact system of a polymer electrolyte membrane, which are based on alkaline exchange membranes.

A comparative study using theoretical computations and electrochemistry of the electroactivity of nickel chalcogenides, specifically NiS, Ni_3_S_2_, and NiS_2_, toward HER in alkaline media has shown that their intrinsic HER activities is in the order of Ni_3_S_2_ (metallic) > NiS_2_ (semiconductor band gap ~0.4 eV) > NiS (semiconductor band gap ~1.1 eV) (Jiang et al., 2016). The origin of the superior performance of Ni_3_S_2_ is the combination of its large electrochemically active surface area (ECSA) and its high conductivity (metallic conductor vs. semiconductor) effects. However, the synthesis of Ni_3_S_2_ particles embedded in nitrogen-doped carbon nanostructures is rarely reported (Jiang et al., 2016; Tian et al., 2019; Wang et al., 2019b; Zhang et al., 2019). In some cases, sulfur powder is introduced as a reactant, which is not very convenient. In addition, there is a lack of fundamental comprehension about the formation of those nickel-sulfur nanostructures. In this contribution, we report a comprehensive study on the development of efficient nanostructured catalysts derived from polyaniline and nickel for the electrocatalytic hydrogen and oxygen evolution reactions (HER and OER) in alkaline media. The introduced thermal treatment enables the assembling of noble metal-free elements of Ni and S into Ni_3_S_2_ particles self-supported on a matrix of C, N, S, and Ni, leading to the construction of carbon nanostructured networks (CNNs). We found that our developed cost-effective nanostructured catalysts allow for a substantial increase in the electrocatalytic performance toward HER (overpotential = 0.19 V at j = −10 mA cm^−2^, Tafel slope = 84 mV dec^−1^) and OER (overpotential = 0.36 V at j = 10 mA cm^−2^, Tafel slope = 35 mV dec^−1^). The results provided by the present study delineate a rational pathway for a further incorporation of additional elements such as Co and Mo as well as the integration into practical electrolyzers.

## Experimental and Methods

### Reagents and Materials

Aniline (ANI, 100%, Alfa Aesar), hydrochloric acid (HCl, 37%, VWR), ammonium persulfate [(NH_4_)_2_S_2_O_8_, 98%, Merck], nickel (II) nitrate hexahydrate [Ni(NO_3_)_2_·6H_2_O, 99%, Acros Organics], potassium hydroxide [KOH, 99.98% (trace metal basis), Acros Organics], isopropanol (iPrOH, 99.5%, Sigma Aldrich), Nafion® suspension (5 wt%, Sigma Aldrich), and commercial catalyst Pt/C (20 wt%, 2 nm, Premetek Co., USA) were used as-received. Nitrogen (N_2_) and oxygen (O_2_) were ultrapure (Air Liquide, France) and all named ultrapure water (MQ) was provided by Milli-Q Millipore source (18.2 MΩ cm at 20°C).

### Synthesis of Polyaniline-Nickel Nanocomposites (PANI-Ni)

PANI-Ni was synthesized by the chemical oxidative polymerization, whereby HCl is the acid doping agent and APS the oxidizing agent. Hundred milliliter solution of HCl (0.5 M) and ANI (0.4 M) was prepared at room temperature by stirring and was transferred in a double-jacket reactor set at 5°C. Another 100 mL solution composed of HCl (0.5 M), APS (0.2 M), and nickel (II) nitrate (0, 0.023 and 0.165 M) was similarly prepared. This solution was added to the previous one under vigorous stirring at 5 mL min^−1^ through a two-syringe infusion pump (KD Scientific). The reaction continued under the aforementioned stirring. After 13 h, the reaction was quenched and the solvent was removed in a rotary evaporator (rotovap) to recover the solid material that was dried in an oven at 80°C overnight. A control trial was run by filtering (Buchner, 0.1–0.2 μm membrane, Whatman) and washing (with ultrapure water until the filtrate is colorless). The thermal stabilization and/or reticulation of the polymer was carried out under air environment (250, 290, 350°C) for 2 h with a heating rate of 2°C min^−1^ in an ashes furnace and materials obtained after this step were referred to as PANI-Ni-TS250, PANI-Ni-TS290, and PANI-Ni-TS350. The calcination was performed under N_2_ in a tubular furnace at 5°C min^−1^ up to the dwell (50°C, 1 h) and slowed down to 2°C min^−1^ toward the target temperature (1,000, 900, 800°C) for a 2, 6, and 12 h stay. A material obtained after this step at 1,000°C was referred to as PANI-Ni-TS290-TC1000 if previously stabilized at 290°C and PANI-Ni-TC1000 when no stabilization had been done.

### Physicochemical Characterization

X-ray diffraction (XRD) patterns were collected in Bragg-Brentano mode from 20 to 80° (in 2θ) using a PANalytical Xpert-PRO diffractometer operating at 40 kV and 20 mA (λ(Cu_Kα1, α2_) = 1.541 Å). Thermogravimetric analysis (TGA) and differential scanning calorimetry (DSC) were performed with a SDT Q600 TA Instruments on aluminum crucibles in the temperature range of 25 to 500°C at 5°C min^−1^ under air atmosphere (100 mL min^−1^). Scanning electron microscopy (SEM) was performed on a Hitachi S-4800 FEG microscope. Energy dispersive X-ray spectroscopy (EDX) was carried out on a ZEISS EVOHD 15 microscope. Brunauer-Emmett-Teller (BET) analysis was performed by the N_2_ adsorption-desorption isotherms (77.3 K) using a Micromeritics ASAP 2020 instrument. CHNS analysis was performed on an Elementar vario MICRO cube. Inductively coupled plasma optical emission spectrometry (ICP-OES) analysis was performed on a spectrometer Optima 2000 DV (PerkinElmer). Raman spectroscopy was performed on a HORIBA Jobin Yvon LabRam HR800UV Raman spectrometer (λ = 659.55 nm) and data were analyzed using pseudo-Voigt functions (LabSpec 5 software). X-ray photoelectron spectroscopy (XPS) characterization was performed on a Thermo Electron ESCALAB 250 spectrometer equipped with a monochromatic radiation source Al Mono (Al_kα_ = 1486.6 eV) operating at 15 kV and 6 mA (survey at a step of 1 eV for transition energy of 150 eV and high-resolution at 0.1 eV for transition energy of 20 eV). The binding energies were corrected on the basis of the energy of C1s at 284.4 eV by using the AVANTAGE software for peaks fitting. The quantification was carried out from the peak area after correction with a suitable sensitivity factor.

### Electrochemical and Catalytic Measurements

The catalytic ink was prepared by mixing 4 mg of the catalytic powder, 360 μL of iPrOH, and 40 μL of Nafion® suspension in a water ultrasonic bath (Elmasonic S 30 H, Grosseron) to form a homogenous ink. The working electrode was a gas diffusion electrode (GDE, AvCarb MGL190, 190 μm thickness, Fuel Cell Earth LL, USA) washed 3 × by iPrOH under middle-shacking and then cut into a L-shape 0.5 cm high and 1 cm wide, leading to an area of 1 cm^2^ (not taking into account the 3D structure of the GDE) and enough space on the top for electrical wiring with gold. Then, 80 μL of catalytic ink was deposed on each side and left to dry at room temperature (50 and 100 μL were used in preliminary steps). All electrochemical tests were fulfilled in a conventional three-electrode cell at 25°C using an AUTOLAB PGSTAT204 potentiostat (Metrohm, Netherlands). A slab of glassy carbon plate (12.4 cm^2^) was used as the counter electrode. Mercury-mercury oxide electrode [Hg|HgO|KOH (0.1 or 1 M), MOE, Origalys] was used as the reference electrode and was separated from the bulk solution by a Haber-Luggin capillary tip. However, the potentials were scaled vs. the reversible hydrogen electrode (RHE) according to the relationship E(V vs. RHE) = E(V vs. MOE) + ΔE. ΔE = 0.920 V (0.1 M KOH) and 0.965 V (1.0 M KOH) at 25°C according to the calibrating curve reported in Supplementary Figure S1. The electrolyte was outgassed with N_2_ before measurements. Electrochemical characterization started with a typical procedure (Houache et al., 2018) that consists of cathodically polarization at E = −0.38 vs. RHE for 5 min and then at E = +0.12 V vs. RHE for 10 min. The electrochemically active surface area (ECSA) was determined from the electrochemical double-layer capacitance (C_dl_) of the catalytic surface using C_s_ = 40 μF cm^−2^ as the average specific capacitance (Trasatti and Petrii, 1991; McCrory et al., 2013): ECSA = C_dl_/C_s_. The electrocatalytic efficiency of the PANI-based electrodes was evaluated toward HER and OER by the method of linear sweep voltammetry (LSV) at 5 mV s^−1^ and the potentiostatic electrochemical impedance spectroscopy (EIS) at different electrode potentials. Accelerated aging tests were carried out by chronoamperometry and chronopotentiometry.

## Results and Discussion

### Effect of the Solvent Removal: Rotovap vs. Buchner Filtration

**Figure 1:**
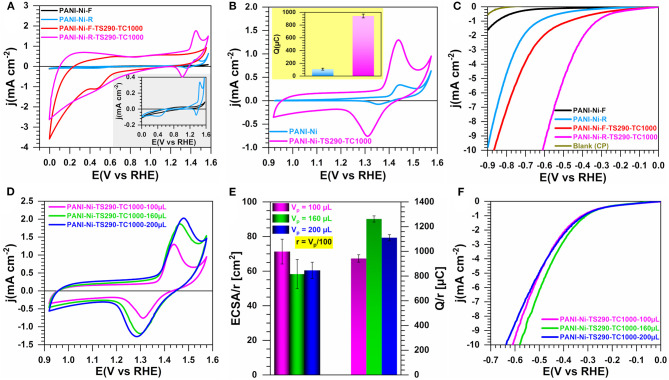
Effect of the solvent removal by filtration (F) or rotovap (R): Electrochemistry in 0.1 M KOH at 25°C. Synthesis with 0.023 M Ni(+II). **(A)** iR-uncorrected steady-state CVs recorded at 50 mV s^−1^ in a large potential window: Inset is CVs of materials without thermal treatment. **(B)** iR-uncorrected steady-state CVs recorded at 50 mV s^−1^ in a reduced potential window: inset is the exchange electrical charge (Q) of the faradaic process of NiO(OH) reduction peak. **(C)** iR-uncorrected HER polarization curves recorded at 5 mV s^−1^. **(D–F)** Optimization of the catalytic ink volume for PANI-Ni-TS290-TC1000 material (rotovap): **(D)** iR-uncorrected steady-state CVs recorded at 50 mV s^−1^; **(E)** Electrochemically active surface area (ECSA) from the double-layer capacitance method (left y-axis) and exchange electrical charge (Q) of the faradaic process of NiO(OH) reduction peak (right y-axis); **(F)** iR-uncorrected HER polarization curves recorded at 5 mV s^−1^. Working electrode is 1 cm^2^ geometric surface area and error bars represent one standard deviation (*n* ≥ 3).

Since our primary goal was to convert the polymerization mixture of ANI, APS, Ni(NO_3_)_2_, HCl, and H_2_O into carbon-nitrogen-sulfur-nickel networks (CNNs), we first examined the effect of the used strategy for the solvent removal on the electrocatalytic properties of the resulting materials. Indeed, nickel being at an ionic state Ni(+II), it is crucial that these species remain trapped in the polymerized material before the thermal treatment at a high temperature of one hundred degrees to get a particle state. Specifically, we compared the synthesis performed with 0.023 M Ni(NO_3_)_2_ and extracted by Buchner filtration (F, 1.5 g recovered) and rotary evaporator (rotovap, R, 8.5 g recovered) methods. For a catalytic ink of 100 μL, Figures 1A,B show the cyclic voltammograms (CVs) recorded at 50 mV s^−1^ for the materials resulting from the filtration (PANI-Ni-F) and rotovap (PANI-Ni-R) together with that obtained after thermal stabilization at 290°C and calcination at 1,000°C for 2 h (PANI-Ni-F-TS290-TC1000 and PANI-Ni-R-TS290-TC1000). It should be noted that, as will be presented in the Section “Effect of the Temperature of Stabilization”, 290°C was chosen as the thermal stabilization as a starting point after consulting its TGA-DSC and on the basis of our own experience in the field of polymer materials stabilization. The presence of the redox peaks between 1.2 and 1.5 V vs. RHE for the samples obtained with the rotovap technique, which belongs to the β-Ni(OH)_2_ + OH^−^ = β,γ-NiO(OH) + e^−^ process (Fleischmann et al., 1971; Alsabet et al., 2011; Houache et al., 2018), indicates the successful incorporation of nickel in the polymerized materials. Based on the synthesis yield and those electrochemical verifications, we argue as a first analysis that the rotovap seems to be the best approach. Furthermore, even if the raw material (PANI-Ni) has the same voltammetric profile as the stabilized and calcined ones, it has however many limitations: (i) a low electrical conductivity, (ii) a possible loss of ionic nickel species during the electrocatalysis, (iii) the remaining of undesired species of chlorine. Hence, the thermal treatment should help to stabilize Ni(+II) is a particle state. Quantitatively, the exchange electrical charge (Q) of the faradaic process of NiO(OH) reduction peak indicated in the inset of Figure 1B is Q = 108 ± 15 and 943 ± 30 μC for PANI-Ni-R and PANI-Ni-R-TS290-TC1000, respectively (see the method in Supplementary Figure S2). Conclusively, even if the low value found for PANI-Ni-R can be justified by a small amount of Ni on the electrode, its direct use remains limited by the aforementioned arguments. To get a clear idea about the electrocatalytic ability, we performed HER on those sets of materials. It should be noted that HER is expected to start at E_eq_ = 0 V vs. RHE in H_2_-satured electrolyte for Pt electrode (Supplementary Figure S1A). Figure 1C shows the iR-uncorrected HER polarization curves recorded at 5 mV s^−1^. Through this manuscript, we will compare the different electrocatalysts by the most used relevant overpotential (η = E-E_eq_) defined as the value corresponding to a current density of |*j*| = 10 mA cm^−2^ (which is the current density expected for a 12.3% efficient solar water-splitting device) (Benck et al., 2014; Luo et al., 2014; Zou and Zhang, 2015). Indeed, during HER or water electrolysis, lots of bubbles are generated on the electrode surface and most of them do not get away from the electrode immediately (this is particularly true for 3D electrodes such as those used as supports herein), which directly decreases the effective active area, and causes an increase of the overpotential (as well as a noise in the polarization curves). So, the overpotential at a high current density cannot be a fair comparison. From Figure 1C, it can be observed that at E = −0.9 V vs. RHE, j = −0.6, −1.6, and −11.7 mA cm^−2^ for CP (support), PANI-Ni-F and PANI-Ni-F-TS290-TC1000, respectively. These results demonstrate that the thermal treatment enables converting the pristine polyaniline into a carbon-nitrogen network with much higher electrocatalytic activity. This positive effect of the thermal treatment is confirmed by comparing PANI-Ni-R and PANI-Ni-R-TS290-TC1000, even though one can logically argue that this is mostly due to the formation of Ni-based particles. Furthermore, the potential at j = −10 mA cm^−2^ is E = −0.86 and −0.61 V vs. RHE for PANI-Ni-F-TS290-TC1000 and PANI-Ni-R-TS290-TC1000, respectively. Therefore, we concluded that the rotovap technique, which will be used for further studies, enables a substantial decrease of the overpotential.

Then, we undertook the optimization of the catalytic ink volume for PANI-Ni-TS290-TC1000 (rotovap), V_p_ = 100, 160 and 200 μL. Figure 1D shows the CVs, which indicate that a volume of 160 μL might be the best compromise since the current associated to the NiO(OH)/Ni(OH)_2_ redox couple is similar for 200 μL. To take into account the amount of nickel, we introduced in Figure 1E the normalization factor r = V_p_/100. This allows us to perform a fair comparison of the normalized values of ECSA determined from the double-layer capacitance method (Supplementary Figure S2) and Q of the faradaic process of NiO(OH) reduction peak. The direct values of ECSA and Q are reported in Supplementary Figure S3, wherein Q = 943 ± 30, 2019 ± 42, 2220 ± 51 μC for 100, 160, and 200 μL, respectively. For the electrocatalytic performance toward HER, Figure 1F displays the iR-uncorrected LSV recorded at 5 mV s^−1^ in 0.1 M KOH electrolyte. Based on this set of data, a catalytic ink volume of 160 μL has been considered for the next steps. It was previously noticed that the heat treatment phase is an essential step in the production of high-performance electrode materials. We then investigated whether the direct calcination without an intermediate step at 290°C would be a better option or not.

### Effect of the Thermal Stabilization

**Figure 2:**
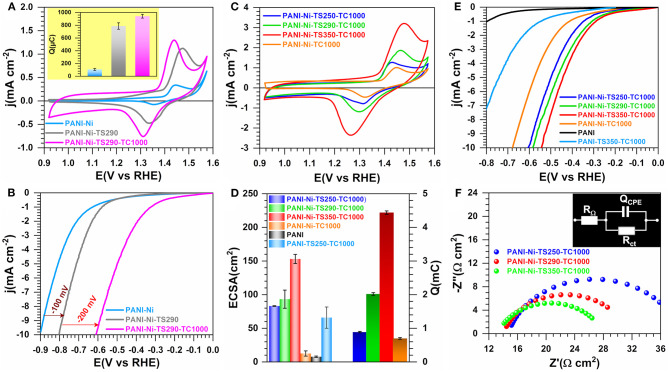
Effect of the stabilization (TS): Electrochemistry in 0.1 M KOH at 25°C. Synthesis with 0.023 M Ni(+II). **(A)** iR-uncorrected steady-state CVs recorded at 50 mV s^−1^ for catalytic ink volume of 100 μL (inset is exchange electrical charge (Q) of the faradaic process of NiO(OH) reduction peak) and **(B)** the corresponding iR-uncorrected HER polarization curves recorded at 5 mV s^−1^. **(C)** iR-uncorrected steady-state CVs recorded at 50 mV s^−1^ for catalytic ink volume of 160 μL. **(C)** iR-uncorrected HER polarization curves recorded at 5 mV s^−1^. **(D)** Electrochemically active surface area (ECSA) from the double-layer capacitance method (left y-axis) and exchange electrical charge (Q) of the faradaic process of NiO(OH) reduction peak (right y-axis). **(E)** iR-uncorrected HER polarization curves recorded at 5 mV s^−1^. **(F)** Complex-plane Nyquist impedance at E_appl_ = −0.43 V vs. RHE (iR-uncorrected): inset shows the equivalent electrical circuit of R_Ω_+*Q*_CPE_//*R*_ct_. Working electrode is 1 cm^2^ geometric surface area and error bars represent one standard deviation (*n* ≥ 3).

The thermal treatment of carbon-based polymers at temperatures of about 200–300°C in air is routine and is the so-called “reticulation step” for the electrospun polyacrylonitrile materials (Wu et al., 2012; Both Engel et al., 2013). To investigate the potential thermal stabilization effect on the electrocatalytic properties, we began a systematic investigation by examining PANI-Ni, PANI-Ni-TS290, PANI-Ni-TS290-TC1000, and PANI-Ni-TC1000. From Figures 2A,B (extended data in Supplementary Figures S4–S6), it is obvious that the number of active sites associated with the redox peaks of NiO(OH)/Ni(OH)_2_ between 1.25 and 1.55 V vs. RHE and the catalytic activity toward HER increase gradually after each thermal step. Since it was found that the thermal stabilization at 290°C is of high importance together with calcination at 1,000°C, we scrutinized lower and higher stabilization temperatures of 250 and 350°C to find out the best condition. Then, the electrochemical performance is compared with a directly calcined material (PANI-Ni-TC1000) and a control material without Ni species, Figures 2C–F. Figure 2C shows that the directly calcined material PANI-Ni-TC1000 has the lowest current density, which is correlated to the values of ECSA (13 ± 4 cm^2^) and Q (699 ± 25 μC) from Figure 2D. Furthermore, a thermal stabilization temperature of 350°C gives the best results in terms of ECSA (153 ± 7 cm^2^) and Q (4.44 ± 0.05 mC), while a temperature of 250°C yields to ECSA = 33 ± 1 cm^2^ and Q = 0.89 ± 02 mC that are lower than 290°C (ECSA = 94 ± 13 and Q = 2.02 ± 0.04). For HER (Figure 2E), the performance trend at j = −10 mA cm^−2^ is PANI-Ni < PANI-Ni-TC1000 < PANI-Ni-TS250-TC1000 < PANI-Ni-TS290-TC1000 < PANI-Ni-TS350-TC1000. This performance trend can be explained by the fact that the stabilization temperature strongly affects the composition of the recovered material, thus on its electrochemical properties. This is further verified by the TGA curve of the PANI-Ni material where the mass loss profile is not the same for the targeted three temperatures of 250, 290, and 350°C. Indeed, in addition to the well-known effect of the temperature on the nature on the stabilization of the carbon-based polymers materials (as explained and referenced above), the temperature also impacts the decomposition of nickel precursor of nitrate/sulfate. Those data imply a significant enhancement of the electrochemical kinetics, which can be translated as a decrease of the charge transfer resistance (R_ct_). This metric, R_ct_, is inversely proportional to the exchange current density (j_0_) and thus to the standard rate constant (k°), and reflects the ability of electron transfer and the difficulty of an electrode reaction for driving a larger current density with a minimum driving force (overpotential). It also gives an idea about the number of electrons that are transferred from the catalytic surface to the reactant(s), as well as intermediate(s) formation inside the double layer. In fact, in heterogeneous electrocatalysis, the reaction occurs near the electrode surface so that the reaction rates depend strongly on the nature of both the electrode material and the electrolyte, i.e., solvated ions in the vicinity of the electrode (from the angstrom to a few nanometers), leading to the appellation of “interfacing electrochemistry” (Bard and Faulkner, 2001; Markovic, 2013; Shao and Markovic, 2016). From the modeling of the “electrode-electrolyte” by the electrochemical impedance spectroscopy, it was found that the resistance to the electrons transfer at this interface is directly impacted by the nature of the reactant(s) as well as intermediate(s) formation inside the double layer. Hence, R_ct_ is expected to be as small as possible in order to get the lowest overpotential. So, to get insights about R_ct_, we performed EIS measurements. Figure 2F shows an example of EIS data at E = −0.43 V vs. RHE (iR-uncorrected). The data were fitted using R_Ω_+*Q*_CPE_//*R*_ct_ as a representative equivalent electrical circuit wherein R_Ω_ is the ohmic resistance (also referred to as cell resistance) that makes the measured overpotential of the electrode larger than the true value and Q_CPE_ is the constant phase element to model “imperfect” capacitors (Orazem and Tribollet, 2017). Supplementary Table S1 gathers all extracted data. At an overpotential of 280 mV, the extracted R_ct_ values are 220, 132, and 104 Ω cm ^2^ for 250, 290, and 350°C, respectively. In addition, the trend in the ohmic resistance is R_Ω_ = 14.9, 14.3, and 13.3 Ω cm^2^ for 250, 290, and 350°C, respectively. Since the cell configuration was kept unchanged, these results show that the initial stabilization plays a further key role in the electrode-electrolyte interface, causing an extra potential drop (R_Ω_) and regulating the electron transfer ability (R_ct_). Even if the polymer reticulation could be one of the reasons, those outcomes could mostly be explained by the formation of Ni-based species that are more affected by the change of the temperature.

**Figure 3:**
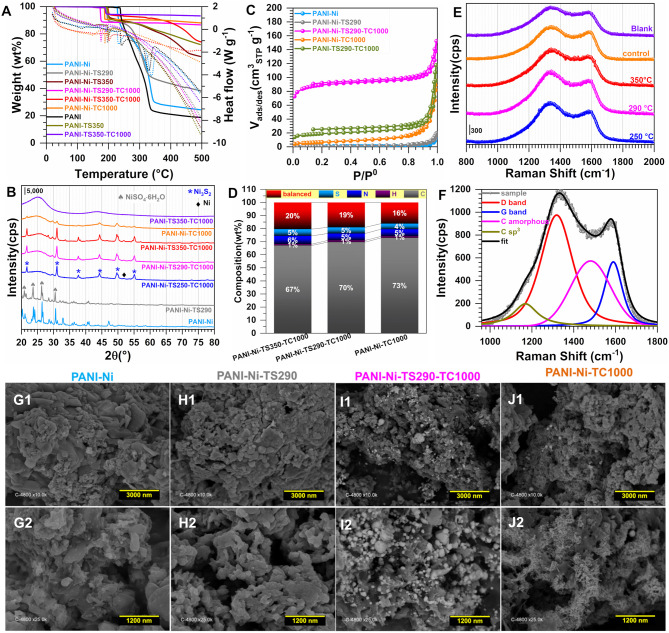
Effect of the stabilization (TS): Physico-chemical characterization. Synthesis with 0.023 M Ni(+II). **(A)** TGA (left y-axis, solid lines) and DSC (right y-axis, dotted lines) curves at 5°C min^−1^ and 100 mL min^−1^ air flow. **(B)** XRD patterns. **(C)** N_2_ adsorption-desorption isotherms (77 K). **(D)** Results from CHNS analysis. **(E)** From bottom to top: Raman spectroscopy of PANI-Ni-TS250-TC1000 (250°C), PANI-Ni-TS290-TC1000 (290°C), PANI-Ni-TS350-TC1000 (350°C), PANI-Ni-TC1000 (control) and PANI-TS350-TC1000 (blank). **(F)** The fitted Raman spectroscopy of PANI-Ni-TS290-TC1000. **(G–J)** Backscattered SEM images of: **(G1,G2)** PANI-Ni, **(H1,H2)** PANI-Ni-TS290, **(I1,I2)** PANI-Ni-TS290-TC1000, **(J1,J2)** PANI-Ni-TC1000.

To gain further insight into the observed difference, we next performed a number of physicochemical characterizations. Figure 3A displays the TGA-DSC curves, based on which the stabilization temperatures have been chosen. Indeed, PANI displays endothermic peaks at 241 and 330°C while the introduction of Ni(NO_3_)_2_ induces a negative shift for the first peak toward 232°C and a positive shift for the second one toward 340°C. This could indicate that those nickel species have an effect on the phenomena occurring there. The absence of any significant weight loss below 150°C would indicate that the materials were well dried, since the evaporation of water molecules that are adsorbed and/or trapped between the hydroscopic polymer chains is expected to occur within this temperature range (Lubentsov and Timofeeva, 1991; Gomes and Oliveira, 2012). Indeed, the elimination of the residual doping agent and the decomposition of smaller polyaniline fragments take place between 200 and 350°C while the degradation of the main PANI chains occurs above 350°C (Wang et al., 2015; Shah et al., 2019). For the materials that were thermally treated at 290, 350, and 1,000°C, the behavior below 200°C is explained by the aforementioned processes. The singularity of the PANI-Ni-TS350-TC1000 material above 350°C is unknown and would need additional deeper experimentation to find out the nature of the lost species. We next utilized XRD to determine the crystallographic structure of the as-synthesized materials. Figure 3B shows the obtained patterns of the representative samples. For PANI-Ni and PANI-Ni-TS290, the peaks at 20.8, 23.4, 26.2, and 30.2° correspond to the (112), (104), (200), and (204) reflections of NiSO_4_·6H_2_O, respectively (Beevers and Lipson, 1932). For the calcined materials, Ni_3_S_2_ has been identified as the main phase with the (101), (110), (003)/(021), (202), (113)/(211), and (122) Bragg peaks appearing at 21.8, 31.2, 37.7/38.2, 44.3, 49.80/50.0, and 55.2°, respectively (JCPDS n° 44-1418) (Wang et al., 2007, 2019b; Zhou et al., 2013a,b; Ou et al., 2014; Jiang et al., 2016; Tian et al., 2019; Zhang et al., 2019). The remaining small peaks could be attributed to a few amounts of other phases of NiS, NiS_2_, and Ni. Ni at bulk or nanoscale is so far known for its excellent performance in alkaline water electrolysis. It was recently reported that Ni_3_S_2_ embedded in different supports exhibits a distinguished electrocatalytic activity, which suggests that the as-prepared materials would be a good candidate for the electrocatalysis (Zhou et al., 2013a; Ou et al., 2014; Jiang et al., 2016; Tian et al., 2019; Wang et al., 2019a,b; Zhang et al., 2019). Those XRD results about the formation of a heterogeneous material explain the above performance trends toward HER. Figure 3C shows the adsorption-desorption isotherms of N_2_ on the different materials; the pore size distribution curves are reported in Supplementary Figure S7 and the extracted quantitative data in Supplementary Table S2. The evaluated specific surface area following the Brunauer-Emmett-Teller (BET) theory is S_BET_ = 4, 4, 302, 24, 65 m^2^ g^−1^ for PANI-Ni, PANI-Ni-TS290, PANI-Ni-TS290-TC1000, PANI-Ni-TC1000, and PANI-TS290-TC1000, respectively. For the same order, the total volume is V_tot_(cm^3^ g^−1^) = 0.031, 0.030, 0.182, 0.146, and 0.162 while the pore radius evaluated from Joyner and Halenda method is R_BJH_(nm) = 32, 52, 12, 27, and 27. These results confirm that the stabilization temperature played a key role in obtaining materials with a high surface area and larger pore volume, which are important for electrocatalysis, as observed in Figure 2. Figure 3D and Supplementary Figure S8 and Supplementary Tables S3, S4 show the CHNS analysis of the synthesized materials. The trend in S amount is PANI-Ni-TC1000 (4.3 wt% S) < PANI-Ni-TS290-TC1000 (4.7 wt% S) < PANI-Ni-TS350-TC1000 (5.2 wt% S), which is supported by the C/S atomic ratio that follows the same trend. This can also explain the above results from XRD and the electrochemical performance. Results from ICP analysis (Supplementary Table S5) confirm the theoretical expectation of about 8 wt% Ni species in the calcined materials, meaning that the exact amount of Ni loading onto the electrode is about 0.1 mg cm^−2^. We further utilized Raman spectroscopy to gain additional insights into the nature of the carbon species present in/on these materials. The results are reported in Figure 3E for the different calcined materials. Figure 3F shows the example of the fitted Raman spectroscopy of PANI-Ni-TS290-TC1000 to extract fundamental data, which are reported in Supplementary Table S6. There are four components. The band at 1,589–1,594 cm^−1^ is assigned to the G-mode, corresponding to the E_2g_ symmetry, and provides information about the in-plane vibration of the sp^2^-bonded carbon atoms (C=C stretching vibrations) (Wang et al., 1990; Nistor et al., 1994; Jawhari et al., 1995; Sadezky et al., 2005). The so-called D mode appears in the 1,311–1,331 cm^−1^ range, also referred to as D-band (A_1g_ symmetry) and results from the symmetry breakdown for carbon atoms situated at the edge of graphite sheets or near defects in the graphene lattice (Wang et al., 1990; Schwan et al., 1996; Pimenta et al., 2007; Ma et al., 2012). While the Raman shift for the G band is nearly the same for the different materials, there is a slight difference for the D band. Finally, the amorphous sp^2^ phase of carbon appears at *ca*. 1,480 cm^−1^ between the bands G and D (Nistor et al., 1994; Jawhari et al., 1995; Sadezky et al., 2005), and the band at about 1,165 cm^−1^ had been successively assigned to nanocrystalline diamond (Schwan et al., 1996), hexagonal diamond (Kohanoff, 1994), and sp^3^ rich phase (Veres et al., 2008). The determined graphitization degree (A_D_/A_G_) allows determining the in-plane crystallite size (L_a_) (Cançado et al., 2006; Ma et al., 2012) for the different materials. That in-plane crystallite size slightly decreases as the temperature of the stabilization increases, from L_a_ = 15.8 nm for PANI-Ni-TS250-TC1000 to L_a_ = 14.6 nm for PANI-Ni-TS350-TC1000. This indicates that the size of sp^2^ clusters presenting a long-range order is affected by those stabilization temperatures.

(1) $$\begin{array}{r} L_{a}\left(nm\right)=\left(2.4\times 10^{-10}\right)\lambda^{4}{\left(\frac{A_{D}}{A_{G}}\right)}^{-1} \end{array}$$

where λ is the laser wavelength in nm, A_D_ is the area of the D band and A_G_ the area of the G band.

**Figure 4:**
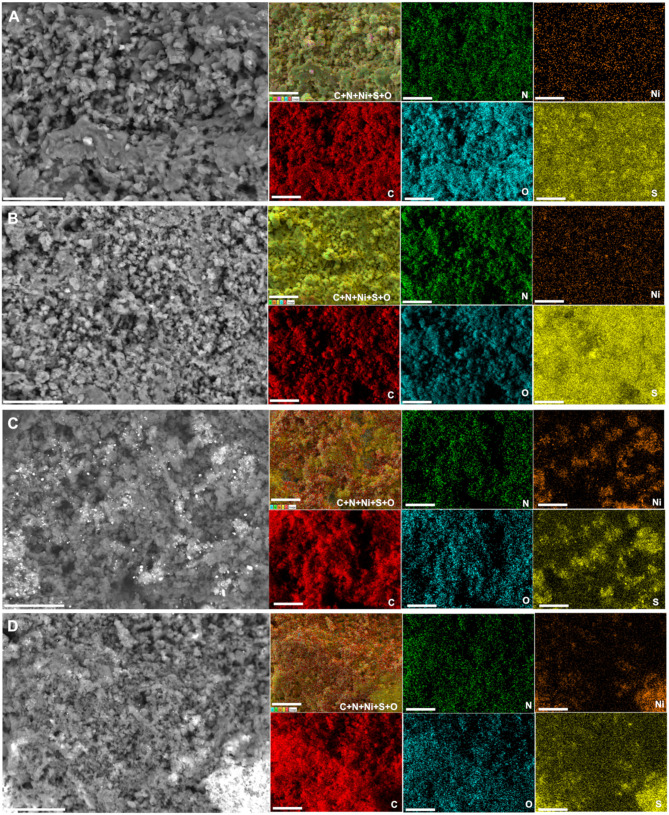
Effect of the stabilization (TS): EDX characterization. Synthesis with 0.023 M Ni(+II). Backscattered SEM images and the corresponding EDX mapping of: **(A)** PANI-Ni, **(B)** PANI-Ni-TS290, **(C)** PANI-Ni-TS290-TC1000, and **(D)** PANI-Ni-TC1000. Scale bar = 25 μm.

We further performed control SEM analysis on representative materials to determine whether the thermal stabilization step can influence the morphology of the obtained polyaniline-based materials. To this end, backscattered SEM images of PANI-Ni, PANI-Ni-TS290, PANI-Ni-TS290-TC1000, and PANI-Ni-TC1000 are shown in Figures 3G–J. It can be clearly observed that no metallic state of nickel particles is formed for the pristine PANI-Ni and stabilized PANI-Ni-TS290. This confirms the previous XRD where nickel was at the sulfate state. Upon the increase of the temperature to 1,000°C, the intermediate material is decomposed into crystalline metallic nickel. We next investigated the distribution of the different elements of C, N, S, O, and Ni during the different stages. Figure 4 shows the obtained EDX mappings from the corresponding backscattered SEM images. The results demonstrate that all the elements are homogeneously distributed when no calcination was done at the high temperature of 1,000°C (Figures 4A,B). Importantly, the mapping of the PANI-Ni-TS290-TC1000 and PANI-Ni-TC1000 materials substantiate the formation of nickel-sulfur compounds (Figures 4C,D), as previously determined by XRD. For those materials, the absence of the Ni and O signals overlapping enables us to completely rule out the significant formation of oxide, hydroxide, or oxyhydrooxide forms of nickel. Quantitative data in terms of the atomic ratios in Supplementary Figure S9 highlight that the experimental Ni/S atomic ratio is about 1.5, thus confirming the formation of Ni_3_S_2_ phase. The results also show the complete removal of chlorine. Furthermore, the concentration of nickel used in the starting material was found to be too low to obtain a well-defined morphology of these Ni_3_S_2_ particles and further provide a rational explanation. But, we postulated that the so-called vapor-liquid-solid (VLS) mechanism might explain the formation of Ni-based nanoparticles. To this end, and also to achieve high catalytic performance for comparison with existing literature, a higher concentration of Ni(+II) of 0.165 M was used in the next sections. Based on ICP results (Supplementary Table S5), Ni loading will increase from 0.1 to 0.5 mg cm^−2^ since for the majority of the reported systems, Ni = 0.5–2 mg cm^−2^ (Feng et al., 2018; Wu et al., 2018; Suryanto et al., 2019). A comparison study is reported in Supplementary Figure S10, which particularly emphasizes the increase of ECSA.

### Effect of the Duration of the Calcination

**Figure 5:**
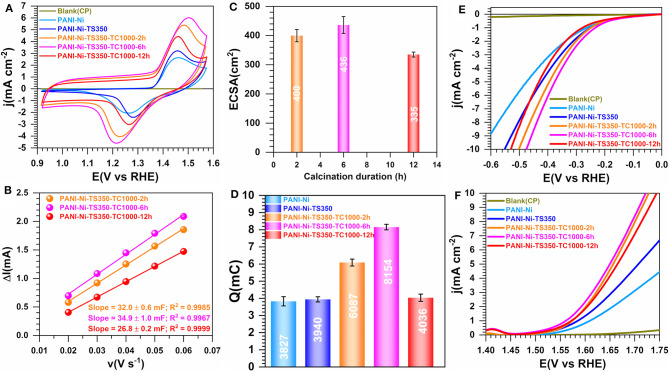
Effect of calcination time (TC): Electrochemistry in 0.1 M KOH at 25°C. Synthesis with 0.165 M Ni(+II). **(A)** iR-uncorrected steady-state CVs recorded at 50 mV s^−1^. **(B)** Plots of the absolute value of the charging current (ΔI = I_a_-I_c_) of the double-layer region at 0.93 V vs. RHE as a function of scan rate for determining the electrochemically active surface area (ECSA). **(C)** ECSA as a function of the duration of the calcination. **(D)** Exchange electrical charge (Q) of the faradaic process of NiO(OH) reduction peak. **(E)** iR-uncorrected HER polarization curves recorded at 5 mV s^−1^. **(F)** iR-uncorrected OER polarization curves recorded at 5 mV s^−1^. Working electrode is 1 cm^2^ geometric surface area and error bars represent one standard deviation (*n* ≥ 3).

The preceding discussion shows that we were able to tightly regulate the formation of Ni_3_S_2_ phase within carbon-nitrogen-sulfur-nickel networks from a thermal treatment of a polyaniline-nickel(II) mixture. The stabilization temperature (TS) of 350°C has been found to be optimal according to Figure 2. We then wondered whether the duration of the calcination step that leads to the formation of this material could be a major factor. To elucidate that point, we further carried out a systematic study to evaluate the impact of the calcination time (TC) of 2, 6, and 12 h. Specifically, the formation of nickel-based particles and the rearrangement of the different atoms leading the bonds such as carbon-carbon (C-C, C=C, C-H), carbon-nitrogen (pyridinic-N, pyrrolic-N, and graphitic-N), and nickel-sulfur depends not only on the value of the calcination temperature, but also on the residence time. Specifically, the duration of calcination is directly related to the time required to form a reducing atmosphere in the furnace and the time needed to form specific type of compounds depending on the nature of the species constituting in the starting sample. Figures 5A–F present the electrochemical study by comparison with control and blank materials CP, PANI-Ni, and PANI-Ni-TS350. Extended data for the evaluation of ECSA are reported in Supplementary Figure S11. The profiles of CVs in Figure 5A (and Supplementary Figure S11A) are similar to those obtained with a lower amount of nickel in the starting material. From Figures 5B–D, the sample produced by the thermal calcination for 6 h has the highest number of active sites (C_dl_ = 35 ± 1 mF, ECSA = 436 ± 21 cm^2^, 8.15 ± 0.16 mC) in comparison to 2 h (C_dl_ = 32 ± 1 mF, ECSA = 400 ± 27 cm^2^, Q = 6.09 ± 0.20 mC) and 12 h (C_dl_ = 27 ± 1 mF, ECSA = 335 ± 9 cm^2^, 4.04 ± 0.21 mC). The electrocatalytic activity toward both HER and OER in 0.1 M KOH electrolyte follows the same tendency (Figures 5E,F). From these results, the decrease in the number of the active sites (quantified by C_dl_, ECSA and Q) together with the performance toward HER and OER when the material was exposed to a longer duration might be explained by the particles sintering. Conclusively, a duration of 6 h appears to be the optimal calcination time for the next steps.

**Figure 6:**
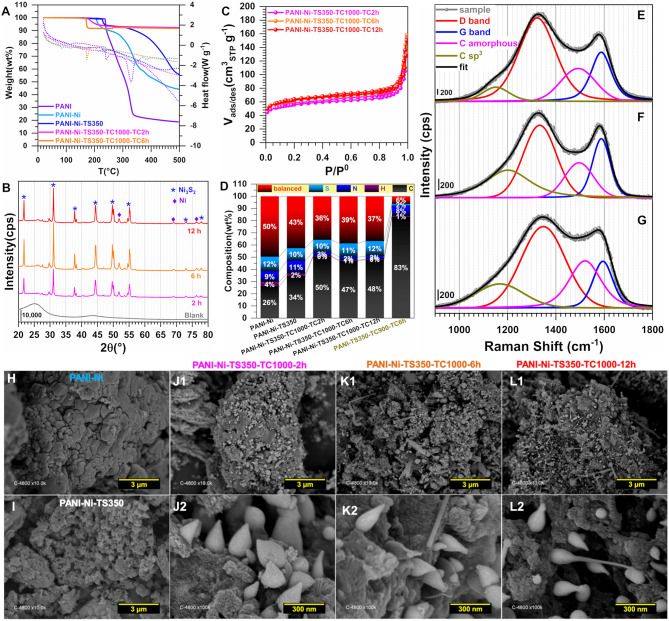
Effect of calcination time (TC): Physico-chemical characterization. Synthesis with 0.165 M Ni(+II). **(A)** TGA (left y-axis, solid lines) and DSC (right y-axis, dotted lines) curves at 5°C min^−1^ and 100 mL min^−1^ air flow. **(B)** XRD patterns. **(C)** N_2_ adsorption-desorption isotherms (77 K). **(D)** Results from CHNS analysis. **(E–G)** Raman spectroscopy of: **(E)** PANI-Ni-TS350-TC1000-TC12h, **(F)** PANI-Ni-TS350-TC1000-TC6h, and **(G)** PANI-Ni-TS350-TC1000-TC2h. **(H–L)** Backscattered SEM images of: **(H)** PANI-Ni, **(I)** PANI-Ni-TS350, **(J1,J2)** PANI-Ni-TS350-TC1000-TC2h, **(K1,K2)** PANI-Ni-TS350-TC1000-TC6h, and **(L1,L2)** PANI-Ni-TS350-TC1000-TC12h.

The materials were further physico-chemically characterized, Figures 6A–**L**. The TGA-DSC curves of Figure 6A are similar to those obtained in Figure 3A, except for the good stability of the materials obtained after the calcination for temperatures higher than 300°C. This is consistent with the hypothesis that the only observed mass loss (8 wt%) would come from the uptake of water and the surface oxidation once left in the ambient air. Figure 6B shows the XRD patterns where the Ni_3_S_2_ (JCPDS n° 44-1418) can be clearly indexed. Other small peaks below 30° belong to other phases of NiS. New distinct diffraction peaks appearing at 2θ = 51.8° and 76.4° for the XRD patterns of the calcined samples can be assigned to (200) and (220) crystal planes of the face-centered-cubic phase of Ni (JCPDS n° 04-0850). This could be mean that the peak at 2θ = 44.4° and initially assigned to the (202) of Ni_3_S_2_ (JCPDS n° 44-1418) could also include the contribution of the (111) crystal plane of Ni (JCPDS n° 04-0850) that is expected to appear at about 44.3°. A possible explanation for this could be the incomplete sulfurization of Ni nanoparticles, presumably because of a potential protection by polyaniline which resists the sulfur ions penetrating into the core material. The N_2_ adsorption-desorption isotherms displayed in Figure 6C look similar. Quantitatively, for the calcination duration of 2, 6, and 12 h, S_BET_ = 190, 210 and 215 m^2^ g^−1^, respectively. As presented above, the increase in BET surface area would likely be due to the removal of more species and the evolution of the particles morphology, which would lead to an increase in the external contact surface area. For that sequence, R_BJH_ is about 16, 14, and 13 nm while the total pore volume is V_tot_(cm^3^ g^−1^) = 0.170, 0.168, and 0.154, thus attesting to the mesoporous character of the material since V_micro_ = 0.025–0.001 cm^3^ g^−1^. The CHNS results are shown in Figure 6D in terms of wt% while Supplementary Figure S12 reports the different atomic ratios of C/N, C/H, C/S, and N/S (Supplementary Table S7 gathers the extended data). While no significant change in the sulfur content appears for any Ni-based materials (10–12 wt% of S), the increase of the calcination duration leads to a decrease of nitrogen weight for the thermally treated samples, PANI-Ni-TS350 (10.8 ± 0.2 wt%) > PANI-Ni-TS350-TC1000-TC2h (3.0 ± 0.1 wt%) > PANI-Ni-TS350-TC1000-TC6h (2.4 ± 0.1 wt%) ≈ PANI-Ni-TS350-TC1000-TC12h (2.5 ± 0.1 wt%). This can be explained by an attachment of S to Ni which is not the case with N, and is particularly true since the blank sample has only 1.6 ± 0.1 wt% of S. So, Ni could catalyze the elimination of N during the cyclization since 8.1 ± 0.1 wt% N was found for the blank. As expected from the XRD results, the presence of S in the final compound is dependent on that of Ni. For the calcined Ni-based materials, the balanced amount of 36–37 wt% includes oxygen of about 3-5 wt%, meaning that the final nickel content is ~30 wt%, which confirms the theoretical expectation. Figures 6E–G show the Raman spectroscopy for the three durations of 2, 6, and 12 h (Supplementary Table S6 gathers the extracted data). It can be qualitatively observed that the fraction of the amorphous sp^2^ phase of carbon appearing at *ca*. 1,480 cm^−1^ between the bands G and D is particularly high for the materials obtained after 2 h (Figure 6G), which means that the duration affects the graphitization. It was also interesting to note that the optimal value of A_D_/A_G_ and thus the in-plane crystallite size L_a_ is found for the material PANI-Ni-TS350-TC1000-TC6h.

**Figure 7:**
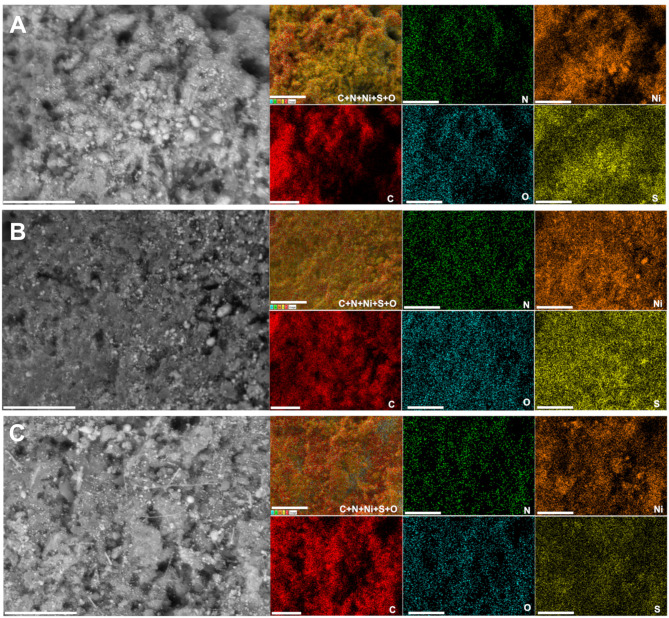
Effect of calcination time (TC): Compositional characterization. Synthesis with 0.165 M Ni(+II). Backscattered SEM images and the corresponding EDX mapping of the material PANI-Ni-TS350-TC1000: **(A)** 2 h, **(B)** 6 h, and **(C)** 12 h. Scale bar = 10 μm.

A deep microscopy analysis was next implemented to account for the morphology of the obtained materials at each stage. Representative backscattered SEM images are displayed in Figures 6H–L (extended images are shown in Supplementary Figures S13–S17). For PANI-Ni and PANI-Ni-TS350, there is no obvious formation of metallic Ni-based particles. After calcination at a high temperature, there is a predominance of cone-shaped micro-scale crystals. A careful observation indicates that the length of the tip increases substantially with the duration of the calcination. Such types of morphology could be explained by a vapor-liquid-solid (VLS) mechanism of growth, involving a vapor of nickel-sulfur so that the super-saturation and nucleation at the liquid/solid interface leads to an axial crystal growth (Wagner and Ellis, 1964). EDX mapping of the different materials were also performed to determine qualitatively the distribution of the elements. Figures 7A–C show the backscattered SEM images and the corresponding EDX maps of C, N, O, S, and Ni. Atomic composition and ratios of C/N, Ni/S, and Ni/O are reported in Supplementary Figure S18 and Supplementary Table S8. It was found that there was no chlorine species after calcination at 1,000°C (about 4 at% Cl in PANI-Ni and 1 at% Cl in PANI-Ni-TS350). After an augmentation from 10.1 ± 0.8 at% to 15.9 ± 0.8 at% after stabilization, the nitrogen content gradually diminished to 3.1 ± 1.0, 2.4 ± 0.4 and 1.9 ± 0.8 at% for 2, 6, and 12 h, respectively. The elimination of nitrogen atoms together with the rearrangement of C-C/C=C bonds at high temperatures to yield compounds of pyridinic-N, pyrrolic-N, and graphitic-N was reported during the carbonization of nitrogen polymers (Wu et al., 2012; Nam et al., 2014; Zhang et al., 2014). XPS analysis in the next sections will elucidate that point. Furthermore, the Ni/S atomic ratios of 1.7 ± 0.4 (2 h), 1.9 ± 0.4 (6 h), and 1.6 ± 0.2 (12 h) are in agreement with the theoretical value of Ni/S = 1.5 in Ni_3_S_2_. Additionally, the mapping of O and Ni elements in Figure 7 and the Ni/O atomic ratio of about 3–4 allow us to completely rule out the possible hypothesis of the formation of NiO, NiO(OH), NiO_2_, or Ni_3_O_4_ species. Based on this electrochemical and physicochemical scrutinizing, the optimal duration for the calcination is 6 h and was used for further studies. Specifically, a screening of the calcination temperature was thereafter carried out to find out the best conditions for the synthesis of a high-performance electrocatalyst.

### Effect of the Temperature of the Calcination

**Figure 8:**
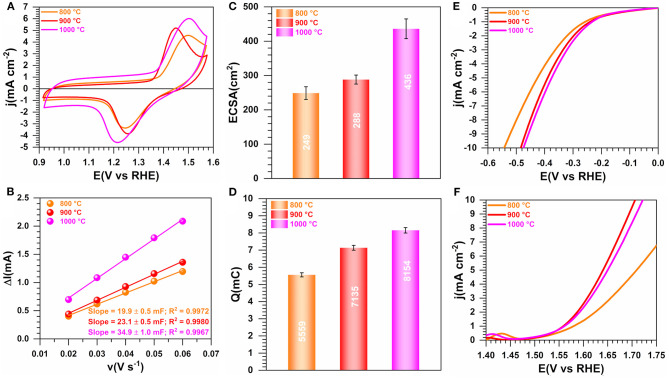
Effect of the calcination temperature (TC): Electrochemistry in 0.1 M KOH at 25°C. Synthesis with 0.165 M Ni(+II). **(A)** iR-uncorrected steady-state CVs recorded at 50 mV s^−1^. **(B)** Plots of the absolute value of the charging current (ΔI = I_a_-I_c_) of the double-layer region at 0.93 V vs. RHE as a function of scan rate for determining the electrochemically active surface area (ECSA). **(C)** ECSA as a function of the temperature of the calcination. **(D)** Exchange electrical charge (Q) of the faradaic process of NiO(OH) reduction peak. **(E)** iR-uncorrected HER polarization curves recorded at 5 mV s^−1^. **(F)** iR-uncorrected OER polarization curves recorded at 5 mV s^−1^. Working electrode is 1 cm^2^ geometric surface area and error bars represent one standard deviation (*n* ≥ 3).

**Figure 9:**
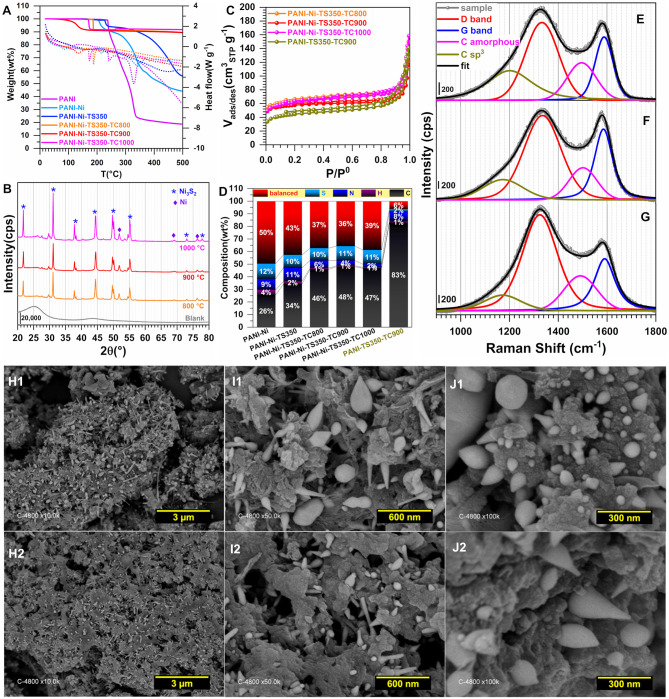
Effect of the calcination temperature (TC): Physico-chemical characterization. Synthesis with 0.165 M Ni(+II). **(A)** TGA (left y-axis, solid lines) and DSC (right y-axis, dotted lines) curves at 5°C min^−1^ and 100 mL min^−1^ air flow. **(B)** XRD patterns. **(C)** N_2_ adsorption-desorption isotherms (77 K). **(D)** Results from CHNS analysis. **(E–G)** Raman spectroscopy of: **(E)** PANI-Ni-TS350-TC1000, **(F)** PANI-Ni-TS350-TC900 and **(G)** PANI-Ni-TS350-TC800. **(H–J)** Backscattered SEM images of: **(H1–J1)** PANI-Ni-TS350-TC900, and **(H2–J2)** PANI-Ni-TS350-TC800.

Two other temperatures of 900 and 800°C were used to elucidate the role of the calcination's temperature on the properties of the materials. Figure 8 shows the obtained electrochemistry results (extended data are reported in Supplementary Figure S19). A better definition of the shape of the voltammetry wave is to be noted in the case of the sample obtained at 900°C, which reflects the electrochemical formation of an oxyhydrooxide NiO(OH) surface followed by the beginning of OER (Figure 8A). Figures 8B,C do not show any significant change of the ECSA when the temperature increases from 800 to 900°C, which is consistent with their voltammetry profiles in the capacitive region (0.9–1.2 V vs. RHE). The drastic change in the faradaic region during the forward going scan translates into a major improvement of Q (Figure 8D) and electrocatalytic activity toward both HER and OER (Figures 8E,F). Specifically, at |j| = 10 mA cm^−2^, E(V vs. RHE) = −0.55, and −0.48 for 800°C and 900/1,000°C during HER and 1.82, 1.72 for 800 and 900/1,000°C during OER, respectively. The significant difference in the case of OER can be explained by the fact that the redox couple NiO(OH)/Ni(OH)_2_ formed during the positive scan above 1.4 V vs. RHE is playing a crucial role. Hence, the presence of S species leads to the electronic regulation of the Ni-S bond that results in an enhanced electrocatalysis, as previously observed (Wang et al., 2007, 2019a,b; Zhou et al., 2013b; Tian et al., 2019; Zhang et al., 2019). Consequently, an operating temperature of 900°C is the optimal value for a better electrocatalytic performance. The physicochemical characterization about the effect of the calcination temperature is shown in Figures 9A–J. Figure 9A shows a slight difference at temperatures lower than 200°C for the sample calcined at 900°C, likely due to its susceptibility to readily adsorb water or oxidize in ambient air. XRD patterns in Figure 9B display the peaks belonging to Ni_3_S_2_ and Ni as previously indexed, the only difference being the intensity of the peaks that augmented with the calcination temperature. From the N_2_ adsorption-desorption isotherms (Figure 8C), which have the same behavior, the determined S_BET_ is 210, 195, and 216 m^2^ g^−1^ for 1,000, 900, and 800°C, respectively (Supplementary Table S2). A comparison with the blank material (PANI-TS350-TC900, S_BET_ = 155 m^2^ g^−1^) highlights an increase of the BET surface that could be attributed to the formed nickel-based crystals, thus creating extra adsorption sites for the probing molecule (N_2_). Elemental analysis results (Figure 9D and Supplementary Table S7) firstly suggest that the nitrogen content diminishes when the temperature increases, N(wt%) = 2.4 ± 0.1, 4.1 ± 0.2, 6.1 ± 0.1 for 1,000, 900, and 800°C, respectively. Secondly, the comparison between PANI-Ni-TS350-TC900 (4.1 ± 0.2 wt% N) and PANI-TC350-TC900 (8.1 ± 0.1 wt% N) undoubtedly shows that the presence of nickel catalyzes the thermal elimination of nitrogen. In the case of sulfur, the reverse is happening with a sulfur removal when nickel is not present in the starting material. This would mean that the majority of the sulfur is not involved in the cycling process. i.e., the cyclization leads to pyridinic-N, pyrrolic-N, and graphitic-N compounds. The remaining S is possibly in the form of thiophene. Hence, all those pyridinic-N, graphitic-N, and thiophene-S species are relevant sources of active sites (Deng et al., 2018). Furthermore, a representative sample (PANI-Ni-TS350-TC900) was analyzed by ICP and the result shows that the Ni content is about 32 wt% (Supplementary Table S5). Taking into account the unavoidable presence of oxygen in the range of 5–7 wt% (Supplementary Table S4), we came up with the balanced result of 37–39 wt%, as shown in Figure 9D.

**Figure 10:**
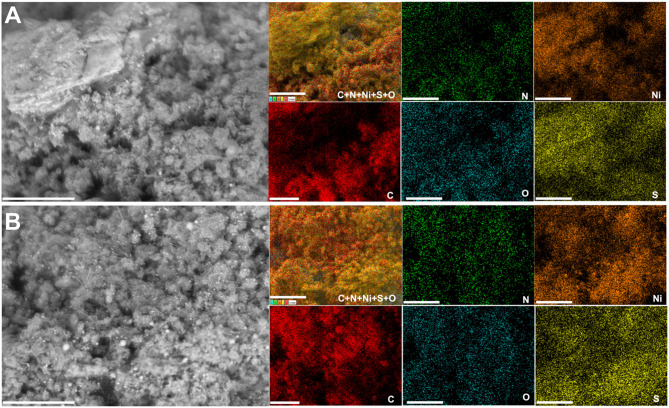
Effect of the calcination temperature (TC): Compositional characterization. Synthesis with 0.165 M Ni(+II). Backscattered SEM images and the corresponding EDX mapping of the material PANI-Ni-TS350-TC6h: **(A)** 800°C and **(B)** 900°C. Scale bar = 10 μm.

Raman spectroscopy fits are shown in Figures 9E–G for PANI-Ni-TS350-TC1000, PANI-Ni-TS350-TC900, and PANI-Ni-TS350-TC800. Those of the blank sample (without nickel) are reported in Supplementary Figure S20. In agreement with the above results, it was found that the temperature of the calcination strongly affects the crystallization of the materials, with A_D_/A_G_ values of 2.1, 2.2, and 2.8 for 1,000, 900, and 800°C, respectively. As a corollary, the in-plane crystallite size is L_a_ = 21.7, 20.9, 16.4 nm for the same sequence. Backscattered SEM images at different magnifications of PANI-Ni-TS350-TC900 and PANI-Ni-TS350-TC800 are shown in Figures 9H–J (extended data in Supplementary Figures S21, S22); those of PANI-Ni-TS350-TC1000 were already shown in Figure 6K and Supplementary Figure S16. The majority of the particles still adopt a conical shape with a heterogeneous crystal size distribution, from tens of nm to hundreds. The above VLS mechanism should hold in the present case. A deeper elemental analysis by EDX (Figures 10A,B) confirms the formation of Ni_3_S_2_ species with co-localization of Ni and S for a Ni/S atomic ratio of 1.88 ± 0.17, and 1.83 ± 0.47 for 900 and 800°C, respectively (Supplementary Figure S18 and Supplementary Table S8). The determined nitrogen content of 2.2 ± 0.5, 4.0 ± 1.2 and 5.2 ± 1.3 wt% for 1000, 900, and 800°C confirms the previous bulk analysis by CHNS, thus underpinning the decrease of nitrogen content upon calcination at high temperatures. This elemental analysis fully substantiates our hypothesis on the regulation of the formation of nickel-based particles and carbon-carbon, carbon-nitrogen, and carbon-sulfur bonds by the duration or the value of the calcination temperature. The previous outcomes demonstrate that we were able to manipulate the synthesis toward different carbon-nitrogen-sulfur-nickel networks possessing various physical and electrochemical properties. Specifically, having demonstrated the ability to use various post-synthesis steps to regulate the growth of Ni_3_S_2_ nanostructured particles from a polyaniline-nickel (II) mixture, we sought to study the electrochemical performance in a relevant concentration of electrolyte (1 M KOH) more carefully and then compare our outcomes with the existing literature. To this end, the optimized material of PANI-Ni-TS350-TC900 was considered.

### Performance in 1 M KOH Electrolyte

**Figure 11:**
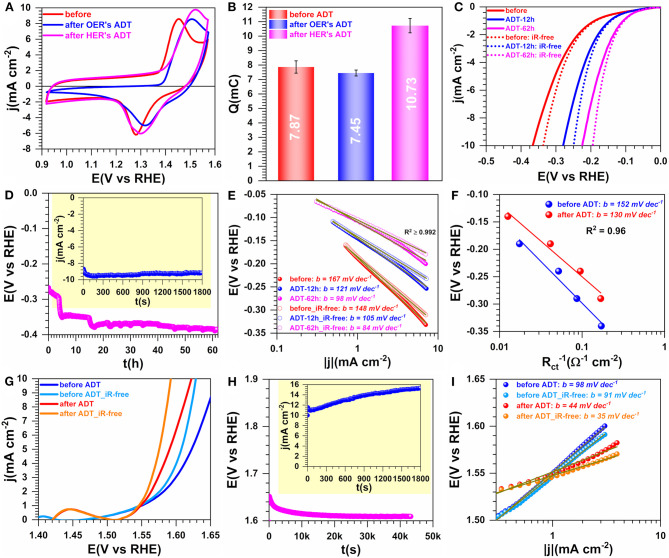
Performance and accelerated aging in 1 M KOH at 25°C: Performance. Synthesis with 0.165 M Ni(+II). **(A)** iR-uncorrected steady-state CVs recorded at 50 mV s^−1^ before and after accelerated durability test (ADT). **(B)** Exchange electrical charge (Q) of the faradaic process of NiO(OH) reduction peak. **(C)** HER polarization curves recorded at 5 mV s^−1^. **(D)** Chronopotentiometry of HER at j = −10 mA cm^−2^ during the ADT: Inset the chronoamperometry of HER at E_appl_ = −0.34 V vs. RHE (iR-uncorrected). **(E)** Tafel plots of HER by the current density. **(F)** Tafel plots by the charge transfer resistance (R_ct_). **(G)** OER polarization curves recorded at 5 mV s^−1^. **(H)** Chronopotentiometry of HER at j = +10 mA cm^−2^ during the ADT: Inset the chronoamperometry of HER at E_appl_ = 1.66 V vs. RHE (iR-uncorrected). **(I)** Tafel plots of HER by the current density. Working electrode is 1 cm^2^ geometric surface area and error bars represent one standard deviation (*n* ≥ 3).

The electrocatalysts conditioning protocol is very crucial for obtaining electrodes with excellent durability. To further evaluate the suitability of the present electrocatalysts for that purpose, we implemented programs of accelerated aging/durability test (ADT) by either potentiostatic or galvanostatic measurements. Figure 11 shows the results obtained by CVs (Figures 11A,B) for HER (Figures 11C–F) and for OER (Figures 11F–I). The charge Q of the faradaic process of NiO(OH) reduction peak increases substantially after HER's ADT by 36%, while a decrease of 5% is observed after OER's ADT. These outcomes can be explained by the simple fact that during HER, either part of the surface of Ni_3_S_2_ will be reduced to pure nickel or still remaining oxidized surface species will be completely reduced to Ni and therefore increase the ECSA. For OER's ADT the occurring oxidation can thereafter lead to a minor loss of the catalytic species (in view of the recorded post-test performance). Specifically, Figures 11C–F show that the efficiency toward HER increases substantially after chronoamperometry at j = −10 mA cm^−2^ with a decrease of the overpotential by *ca*. 150 mV. The determined Tafel slope by the two methods (current density and charge transfer resistance) also decreases significantly. The same trend is observed for OER. The Tafel slope value giving important information on the rate determining step (rds) in an electrochemical reaction is a mechanism descriptor (P. N. Ross et al., 2003). The obtained values of 84–160 mV dec^−1^ suggest that the *rds* of the electrocatalytic process is the so-called “Volmer reaction,” characterized by the limitation from the water molecules adsorption.

Supplementary Figure S23 reports the comparison with the commercial Pt/Vulcan for both HER and OER. The comparative performance with the relevant Ni-based catalysts from the literature in alkaline media is shown in Supplementary Tables S9, S10. At the present PANI-derived N_3_S_2_ catalysts, the overpotential at 10 mA cm^−2^ is 194 mV for HER (Tafel slope = 84 mV dec^−1^) and 360 mV for OER (Tafel slope = 35 mV dec^−1^). In comparison with Pt/Vulcan, the performance is lower for HER (overpotential = 92 mV and Tafel slope = 49 mV dec^−1^) and larger for OER (overpotential = 435 mV and Tafel slope = 62 mV dec^−1^). For similar Ni_3_S_2_-based catalysts, the HER's overpotential is typically 350-474 mV (Jiang et al., 2016; Wang et al., 2019a), which means that the present Ni_3_S_2_ has a higher activity. Even though the overall HER and OER performance is higher than most Ni_3_S_2_-based materials (Oluigbo et al., 2019; Xiao et al., 2019), it is still lower than other reported Ni-based systems involving additional catalytic elements, for instance P, Cu, Fe, Ru, Co, etc., and mostly when the catalytic ink was loading onto nickel foams (Jung et al., 2016; Wang et al., 2016b, 2019a; Hao et al., 2019; Suryawanshi et al., 2019; Yu et al., 2019; Li et al., 2020). Having performed a thorough and step-by-step study of the synthesis conditions and shown that the as-prepared materials exhibit good activity and stability, the remaining opened question we finally sought to address more carefully was the evolution of the oxidation state of the involved elements as well as the surface composition. This is especially important since catalysis is related to the surface atoms. For that, we utilized XPS, a technique which analyzes only a nanoscale domain of ~10 nm penetration since the bulk and semi-bulk analysis have been previously fixed by ICP, CHNS, and EDX.

### XPS Investigation

**Figure 12:**
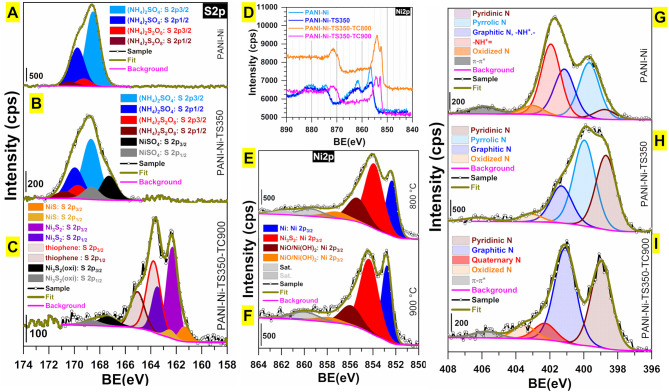
Synthesis with 0.165 M Ni(+II): Compositional and structural characterization by XPS. High-resolution spectra of **(A–C)** S 2p, **(D–F)** Ni 2p and **(G–I)** N 1s for: **(A,G)** PANI-Ni, **(B,H)** PANi-Ni-TS350, and **(C,I)** PANI-Ni-TS350-TC900. **(D)** Overall comparison of Ni 2p. **(E)** Ni 2p for PANI-Ni-TS350-TC800. **(F)** Ni 2p for PANI-Ni-TS350-TC900.

We finally carried out XPS analysis for unraveling the evolution of the surface chemical states and the structural surface composition. Figures 12A–I shows the high-resolution spectra of S 2p, N 1s, and Ni 2p regions. The indexed survey XPS of the as-synthesized materials (PANI-Ni, PANI-Ni-TS350, PANI-Ni-TS350-TC800, PANI-Ni-TS350-TC900, and blank PANI-TS350-TC900) are reported in Supplementary Figure S24. Extended comparative spectra are displayed in Supplementary Figures S25–S28. When no calcination was done, the examination of the XPS of S 2p core-level (Figures 12A,B) reveals the remaining polymerization reactant (NH_4_)_2_S_2_O_8_ at BE = 168.5 eV (S 2p_3/2_) and 169.8 (S 2p_1/2_), the side-product (NH_4_)_2_SO_4_ at BE = 169.3 eV (S 2p_3/2_) and 170.4 (S 2p_1/2_) as well as the formed NiSO_4_ at BE = 167.29 eV (S 2p_3/2_) and 168.65 (S 2p_1/2_) (Yu et al., 1990). After the calcination (Figure 12C and Supplementary Figure S26), all those peaks disappear completely with the emergence of new peaks corresponding to compounds NiS (161.4 eV for S 2p_3/2_ and 162.5 eV for S 2p_1/2_), Ni_3_S_2_ (162.3 eV for S 2p_3/2_ and 162.5 eV for S 2p_1/2_), and thiophene (163.8 eV for S 2p_3/2_ and 165.0 eV for S 2p_1/2_); the peaks at 167.39 and 168.65 eV belong to the oxidized forms of Ni_3_S_2_ (Yu et al., 1990; Park et al., 2015; Jiang et al., 2016; Deng et al., 2018). The presence of Ni_3_S_2_ is further confirmed after examining the XPS of Ni 2p with the peak at 854.3 eV (Ni 2p_3/2_) (Yu et al., 1990; Park et al., 2015; Jiang et al., 2016). The peak at 852.8 eV is assigned to the Ni 2p_3/2_ of the pure Ni phase, as previously observed by XRD. The remaining peaks at 855.9, 857.6, and 860.1 eV correspond to the surface oxidized nickel and satellites (Grosvenor et al., 2006; Biesinger et al., 2009, 2011). For the C 1s (Supplementary Figure S25), the peak at ~285.2 eV is assigned to C-C function and those situated at 286.0 and 288.0 eV are attributed to the C-N/-C-O and carbonyl (C=O) functions, respectively (Andersson et al., 2002; Rajagopalan and Iroh, 2003; Stankovich et al., 2007; Kumar et al., 2016). The N1 s (Figures 12G–I and Supplementary Figure S28) shows the presence of the imine (-N=C, pyridinic N) at ca. 398.8 eV, the amine (-NH-C, pyrrolic N) at ~399.7 eV, the graphitic/polaron/bipolaron at 401.2 eV, the quaternary/-NH^+^= at 402.0 eV, the oxidized nitrogen at 403.0 eV, and the π → π^*^ shake-up satellite of the doublet present on some nitrogen at 405.9 eV (Keane et al., 1991; Yue and Epstein, 1991; Soto et al., 2001; Rajagopalan and Iroh, 2003; Sreedhar et al., 2006; Biddinger et al., 2009; Lai et al., 2012; Tantawy et al., 2015; Deng et al., 2016; Smolin et al., 2017; Inagaki et al., 2018).

**Figure 13:**
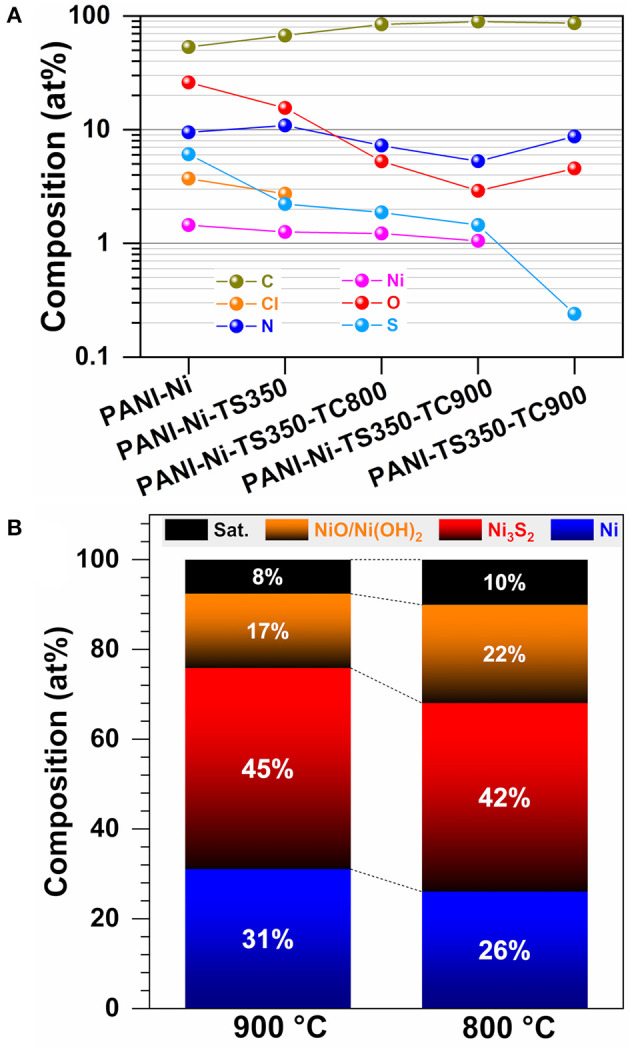
Synthesis with 0.165 M Ni(+II): Compositional and structural characterization by XPS. **(A)** Overall surface atomic composition. **(B)** Surface atomic composition of Ni-based species as-determined from XPS of PANI-Ni-TS350-TC900 and PANI-Ni-TS350-TC800.

The overall atomic composition (surface) is shown in Figure 13A. Specifically, the S/Ni atomic ratio is 4.2, 1.8, 1.5, and 1.4 for PANI-Ni, PANI-Ni-TS350, PANI-Ni-TS350-TC800, and PANI-Ni-TS350-TC900, respectively. Hence, based on the theoretical expected S/Ni ratio of 0.67 in Ni_3_S_2_, we postulated a core-shell like structure for those particles. This is supported by the fact that the majority of the as-synthesized nickel-sulfur particles have a size larger than tens of nanometers and XPS probes a depth of about 10 nm. This hypothesis is also in agreement with the suggested vapor-liquid-solid (VLS) mechanism to account for the formation of those Ni-based particles. Indeed, the sulfur content is 1.45 and 0.24 at % for the samples calcined at 900°C in the presence and absence of nickel, which would mean that at 900°C an atmosphere of sulfurous species is present in the oven and could redeposit onto Ni particles. This hypothesis explained the surprising increase of the electrocatalytic activity during the accelerated aging test. Furthermore, the evaluated surface atomic composition of Ni-based species **(Figure 13B**) indicates that the calcination temperature of 900°C leads to a decrease of surface nickel oxides. For the calcined materials, Supplementary Figure S29A shows that the total nitrogen content of 5.3–8.7 at % is involved in the majority of the pyridinic-N (38–44%) and the graphitic-N (40–46%), which are well-known as the main active nitrogen species, and could also explain the obtained electrocatalytic performance of the materials synthesized herein (Lai et al., 2012; Silva et al., 2013; Deng et al., 2018; Inagaki et al., 2018; Wang et al., 2019c). The presence of pyridinic nitrogen is supported by the atomic distribution of carbon species (SupplementaryFigure S29B) that shows that C=C represents 58–68%.

## Conclusions

Owing to its large natural abundance and involvement in [NiFe] hydrogenases, nickel has long been examined as a promising candidate for hydrogen production catalysis, particularly under alkaline conditions. The present work reports on the use of polyaniline and nickel nitrate as sacrificial supports for the synthesis of self-supported nickel sulfide Ni_3_S_2_ particles onto nitrogen-sulfur-nickel-carbon nanostructured networks for the electrocatalytic hydrogen evolution reaction (HER) and oxygen evolution reaction (OER) in alkaline media. We have integrated different physicochemical (TGA-DSC, Raman spectroscopy, XRD, SEM, EDX, ICP, CHNS, BET, and XPS) and electrochemical (voltammetry and electrochemical impedance spectrometry, EIS) methods to unravel the properties and efficiency of those nanocatalysts. It was observed that an intermediate stabilization step around 350°C in air is a crucial step for the recovery of highly active and durable catalysts upon the calcination at 800–1,000°C under nitrogen gas. The formation of the Ni_3_S_2_ phase was confirmed by both complementary EDX mapping and XRD characterizations. Further SEM studies have shown that the majority of those Ni_3_S_2_ particles from tens of nm to hundreds exhibit a conical shape, which suggest the involvement of the so-called vapor-liquid-solid (VLS) mechanism. Deeper surface state analysis by XPS has demonstrated that the atomic distribution of the elements Ni and S in Ni_3_S_2_ would be of the core-shell type with S-surface enrichment. For the as-synthesized polyaniline-derived Ni_3_S_2_, the electrocatalytic studies in KOH 1 M electrolyte have demonstrated that the overpotential needed to reach the metric current density of 10 mA cm^−2^ is 194 mV for HER (Tafel slope = 84 mV dec^−1^) and 360 mV for OER (Tafel slope = 35 mV dec^−1^) after 24 h of continuous operation. Instead of decreasing, it was surprisingly found that the performance of the catalyst increased during the accelerated aging measurement. To date, the present performance is higher than similar Ni_3_S_2_-based catalysts reported in the literature where the HER's overpotential is typically 350–475 mV at 10 mA cm^−2^. Those results are satisfactory when compared to the commercial Pt/Vulcan benchmark catalyst tested under our conditions toward HER (overpotential = 92 mV and Tafel slope = 49 mV dec^−1^) and OER (overpotential = 435 mV and Tafel slope = 62 mV dec^−1^). Designed to mimic the activity of a [NiFe] hydrogenase biocatalyst that contains at least one Ni atom, the present nickel sulfides of Ni_3_S_2_ constitute an attractive type of bio-inspired promising platinum group metal-free (PGM-free) catalysts for catalytic hydrogen production. Our outcomes are in favor of transitioning from noble-metal-based catalysts to earth-abundant inorganic catalysts, due to the scarcity of those PGMs associated with their high price making them impractical for world-wide hydrogen production applications.

## Data Availability Statement

The data that support the plots within this paper and other findings of this study are available from the corresponding author upon reasonable request.

## Author Contributions

RD, YH, AM, ST, and DCor conceived the project and designed the experiments. YH, AM, ST, and DCor supervised the study. RD and M-AL performed the catalysts synthesis. RD and NM carried out TGA-DSC measurements. VF performed XPS analysis. RD carried out electrochemical measurements. DCot and BR carried out SEM and EDX measurements. RD, PH, and YH performed Raman spectroscopy analysis. AL and JC performed and analyzed XRD measurements. All the authors discussed the results and contributed in the manuscript editing.

## Conflict of Interest

The authors declare that the research was conducted in the absence of any commercial or financial relationships that could be construed as a potential conflict of interest.
