# Supplementary material for: Nanostructured Carbon-Nitrogen-Sulfur-Nickel Networks Derived From Polyaniline as Bifunctional Catalysts for Water Splitting
Source: Front Chem. 2020 May 20;8:385. doi: 10.3389/fchem.2020.00385 (PMC7251167; doi:10.3389/fchem.2020.00385)
Supplement: Supplementary file 1 [file Data_Sheet_1.pdf]

Supplementary Material

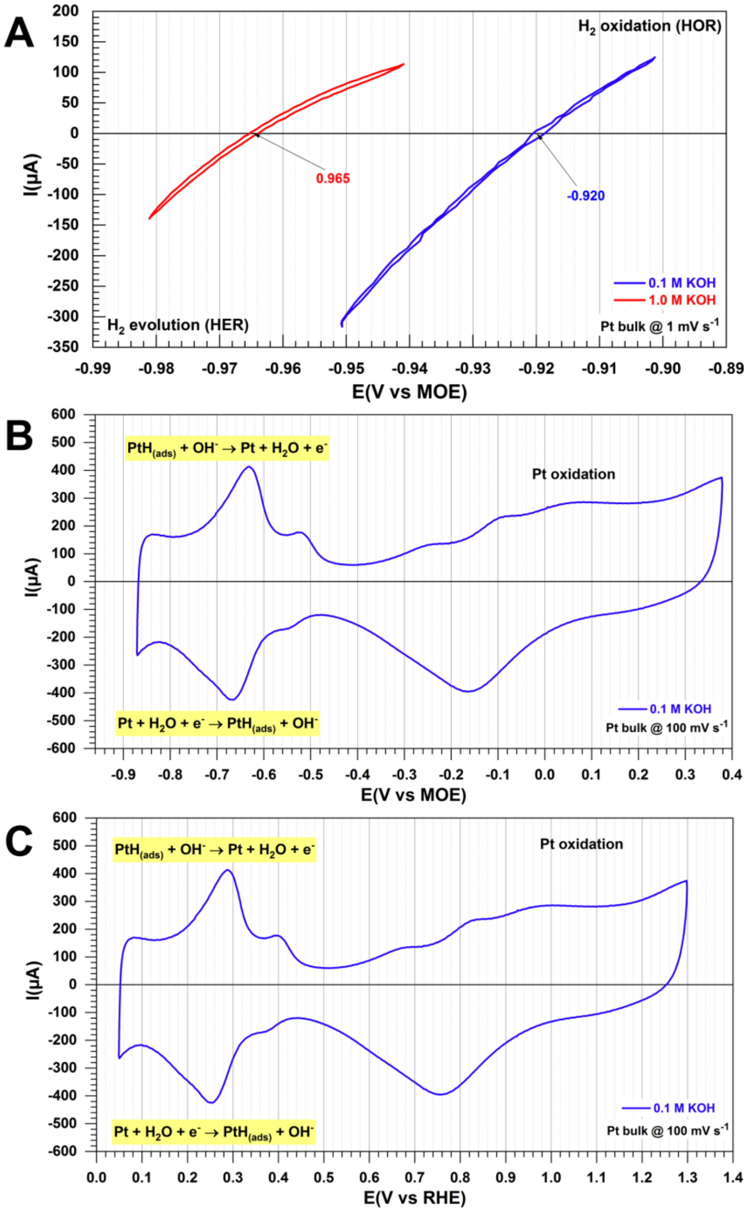

Supplementary Figure S1| (A) Steady-state CVs recorded in H_2-saturated KOH aqueous electrolyte at 1 mV s^{-1} at 25 °C (working electrode = Pt plate, counter electrode = Pt mesh, reference electrode = Hg|HgO|KOH (0.1 M or 1.0 M), referred to as MOE). Note: experiments were performed in a gently stirred solution. **(B, C)** Steady-state CV recorded in N_2-saturated 0.1 M KOH at 100 mV s^{-1} at 25 °C for: **(B)** MOE reference electrode scale and **(C)** RHE reference electrode scale.

<u>Results</u>: The average of the two potentials at which the current crossed zero is taken to be the thermodynamic potential for the hydrogen electrode reactions. Thus, the scaling relationship is: E(V vs RHE) = E(V vs MOE) + x V, x = 0.920 for 0.1 M KOH and x = 0.965 for 1.0 M KOH.

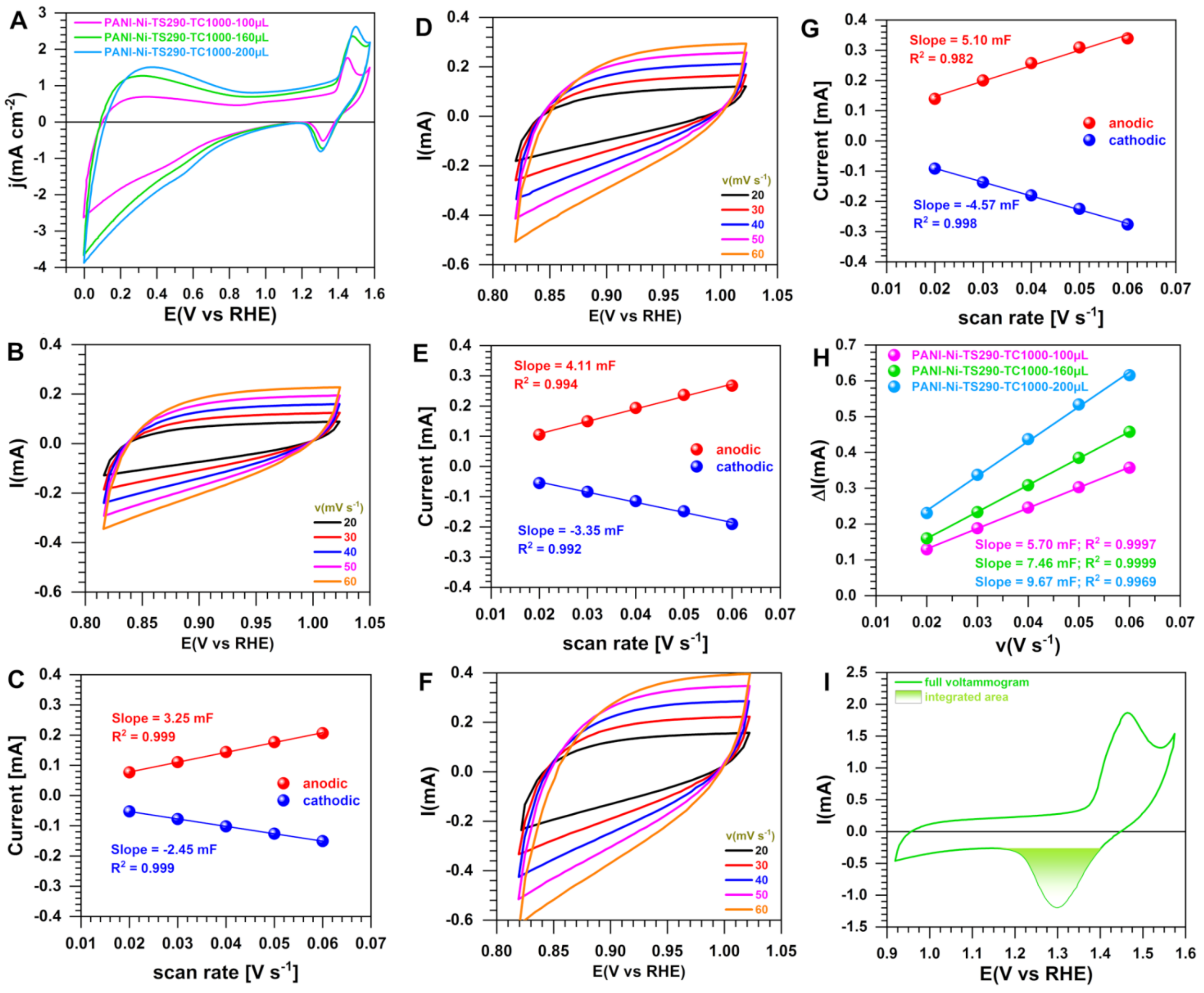

Supplementary Figure S2| Effect of the solvent removal: Electrochemistry in 0.1 M KOH at 25 °C. Synthesis with 0.023 M Ni(+II). (A) iR-uncorrected steady-state CVs recorded at 50 mV s^{-1} in a large potential window. **(B-H)** Double-layer capacitance measurements for determining electrochemically active surface area (ECSA): **(B, D, F)** iR-uncorrected CVs recorded at different scan rates in the double-layer capacitance region and **(C, E, G)** The anodic (I_a) and cathodic (I_c) charging currents measured at 0.93 V vs RHE plotted as a function of scan rate; **(B, C)** PANI-Ni-TS290-TC1000-100µL, **(D, E)** PANI-Ni-TS290-TC1000-160µL and **(F, G)** PANI-Ni-TS290-TC1000-200µL. **(H)** The plots of the absolute value of the charging current ($\Delta I = I_a - I_c$). **(I)** Illustration of the method used for the exchange electrical charge ($Q = \frac{1}{v}\int IdE$) of the faradaic process β-$Ni(OH)_2$ + OH^- →β,γ-NiO(OH) + e^-: The line represents the complete steady-state CV whereas the shaded curve shows the integrated region for the PANI-Ni-TS290-TC1000-160µL.

<u>Results</u>: The double-layer capacitance of the system (C_{dl}) is taken as either the average of the absolute value of the slopes of the linear fits to the data of **Figures S2C, E, G** or the half of the slope of the linear fit to the data of **Figure S2H**. The electrochemically active surface area is then ECSA = C_{dl}/C_s where C_s = 40 µF cm^{-2} and the roughness factor (rugosity) is R_F = ECSA/S_g where S_g = 1 cm^2.

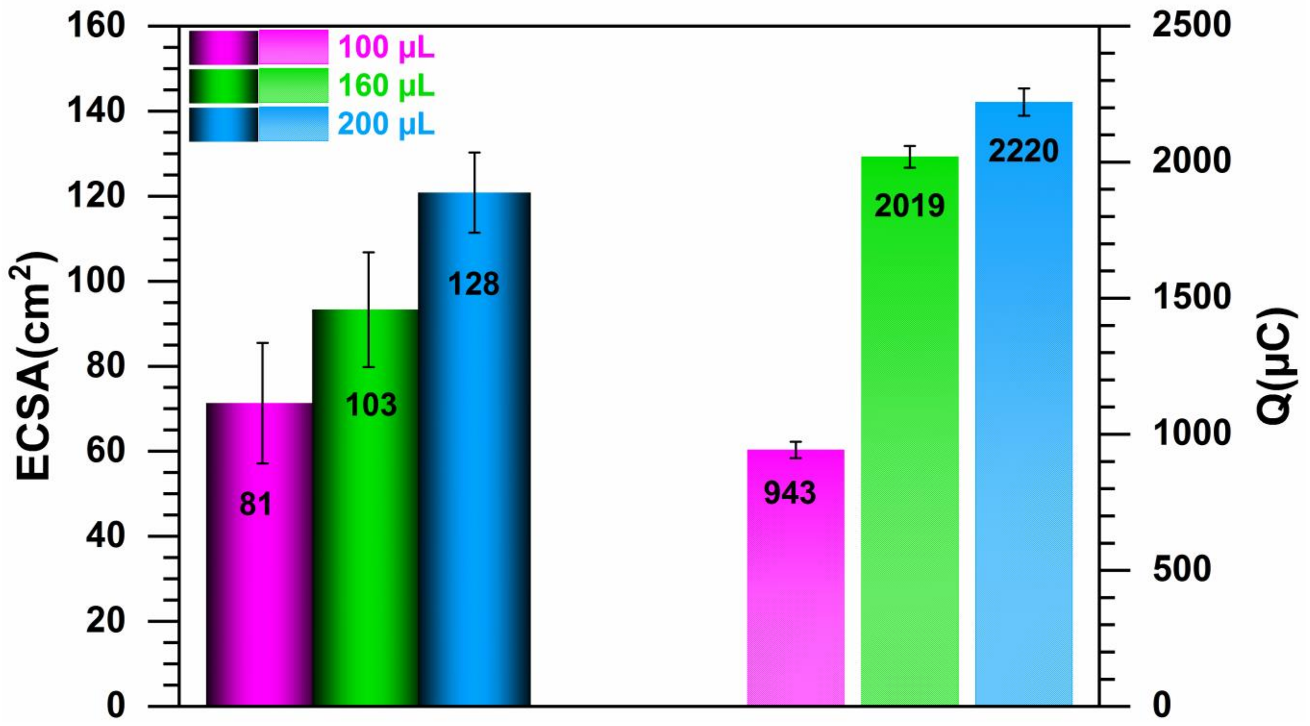

Supplementary Figure S3| Effect of the solvent removal: Electrochemistry in 0.1 M KOH at 25 °C. Synthesis with 0.023 M Ni(+II). Left y-axis: Electrochemically active surface area (ECSA) from the double-layer capacitance method. Right y-axis: Exchange electrical charge (Q) of the faradaic process β-$Ni(OH)_2$ + OH^- →β,γ-NiO(OH) + e^-. Error bars represent one standard deviation ($n \geq 3$).

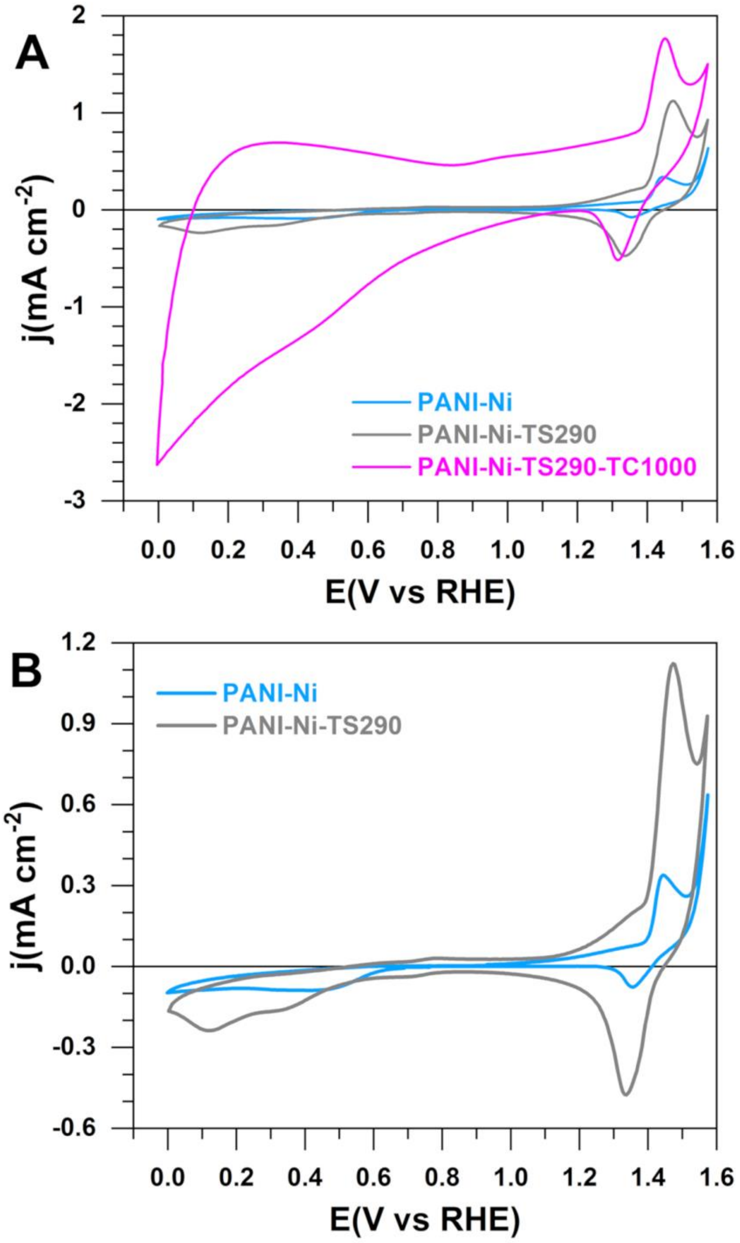

Supplementary Figure S4| Effect of the Thermal Stabilization: Electrochemistry in 0.1 M KOH at 25 °C. Synthesis with 0.023 M Ni(+II). (A) iR-uncorrected steady-state CVs recorded at 50 mV s^{-1} for catalytic ink volume of 100 µL.

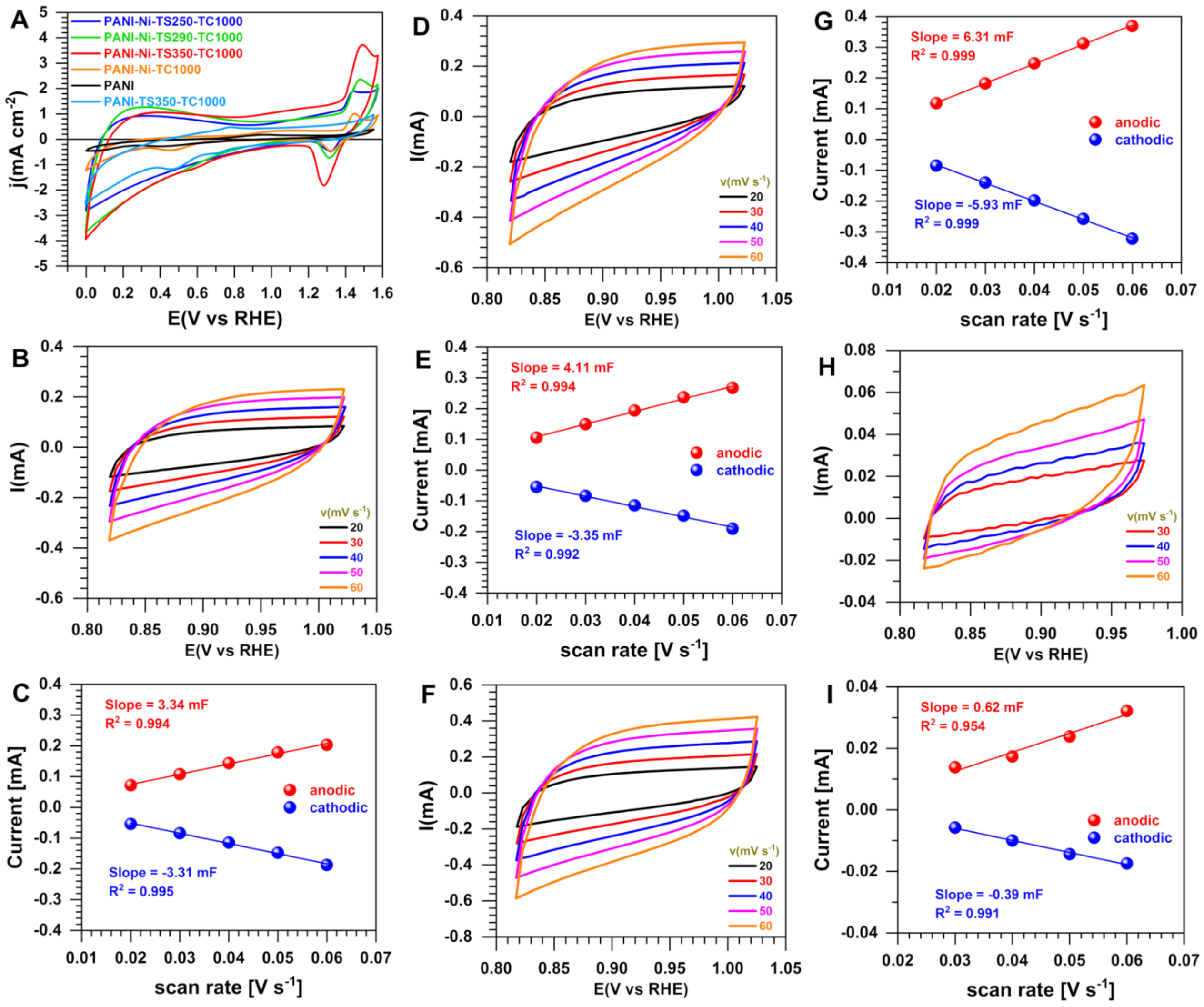

Supplementary Figure S5| Effect of the Thermal Stabilization: Electrochemistry in 0.1 M KOH at 25 °C. Synthesis with 0.023 M Ni(+II). (A) iR-uncorrected steady-state CVs recorded at 50 mV s^{-1} in a large potential window. **(B-I)** Double-layer capacitance measurements for determining electrochemically active surface area (ECSA): **(B, D, F, H)** iR-uncorrected CVs recorded at different scan rates in the double-layer capacitance region and **(C, E, G, I)** The anodic (I_a) and cathodic (I_c) charging currents measured at 0.93 V vs RHE (0.85 V vs RHE for PANI-Ni-TC1000) plotted as a function of scan rate; **(B, C)** PANI-Ni-TS250-TC1000, **(D, E)** PANI-Ni-TS290-TC1000, **(F, G)** PANI-Ni-TS350-TC1000, **(H, I)** PANI-Ni-TC1000.

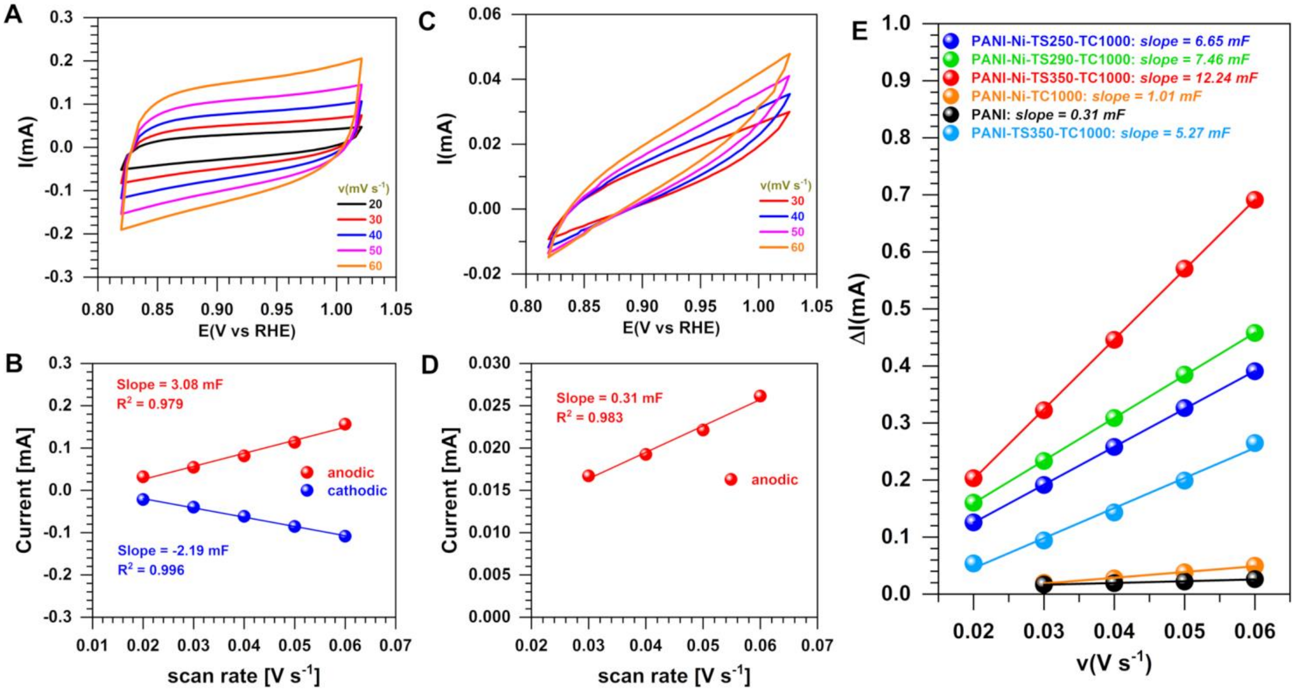

Supplementary Figure S6| Effect of the Thermal Stabilization: Electrochemistry in 0.1 M KOH at 25 °C. Synthesis with 0.023 M Ni(+II). Double-layer capacitance measurements for determining electrochemically active surface area (ECSA): **(A, C)** iR-uncorrected CVs recorded at different scan rates in the double-layer capacitance region and **(B, D)** The anodic (I_a) and cathodic (I_c) charging currents measured at 0.93 V vs RHE plotted as a function of scan rate; **(A, B)** PANI-TS350-TC1000, **(C, D)** PANI. **(E)** The plot of the absolute value of the charging current ($\Delta I = I_a - I_c$).

<u>Note</u>: For the sample PANI, only the anodic part is considered given CV profile of that material in **Figure S5C**.

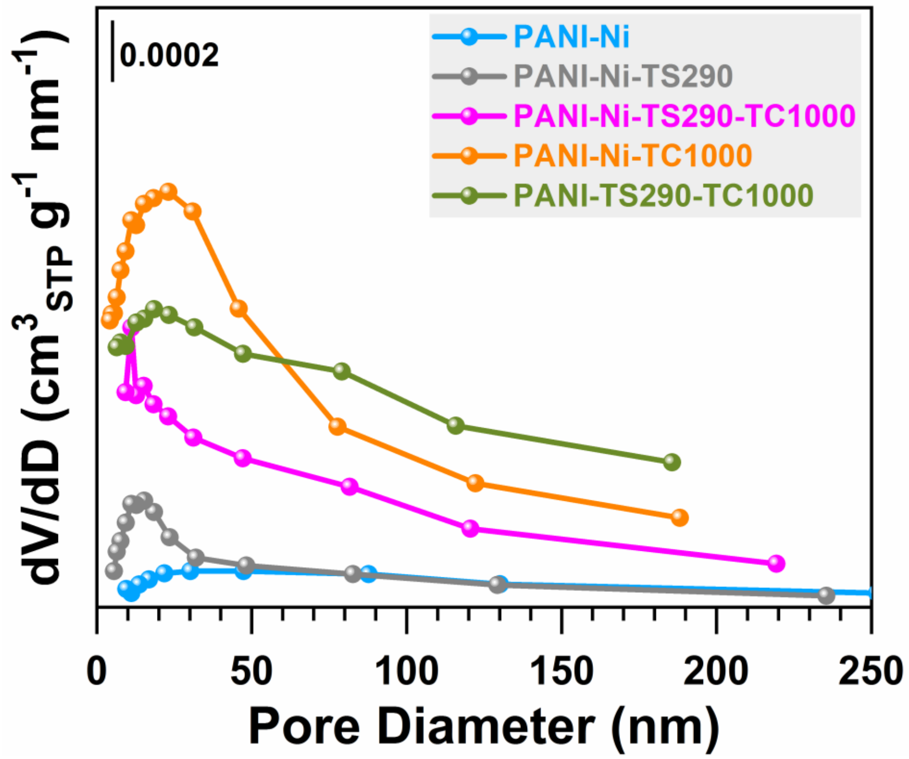

Supplementary Figure S7| Effect of the Thermal Stabilization: Physico-chemical characterization. Synthesis with 0.023 M Ni(+II). The corresponding pore size distribution curves from the N_2 adsorption-desorption isotherms (77 K).

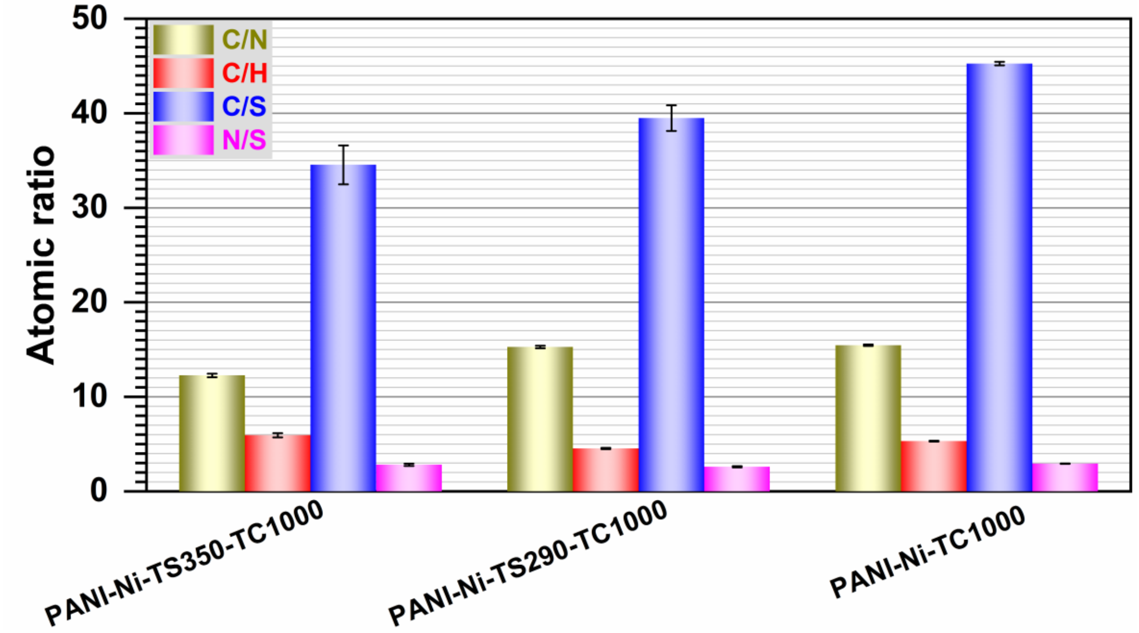

Supplementary Figure S8| Effect of the Thermal Stabilization: Physico-chemical characterization. Synthesis with 0.023 M Ni(+II). Results from CHNS analysis. Error bars represent one standard deviation ($n \geq 3$)

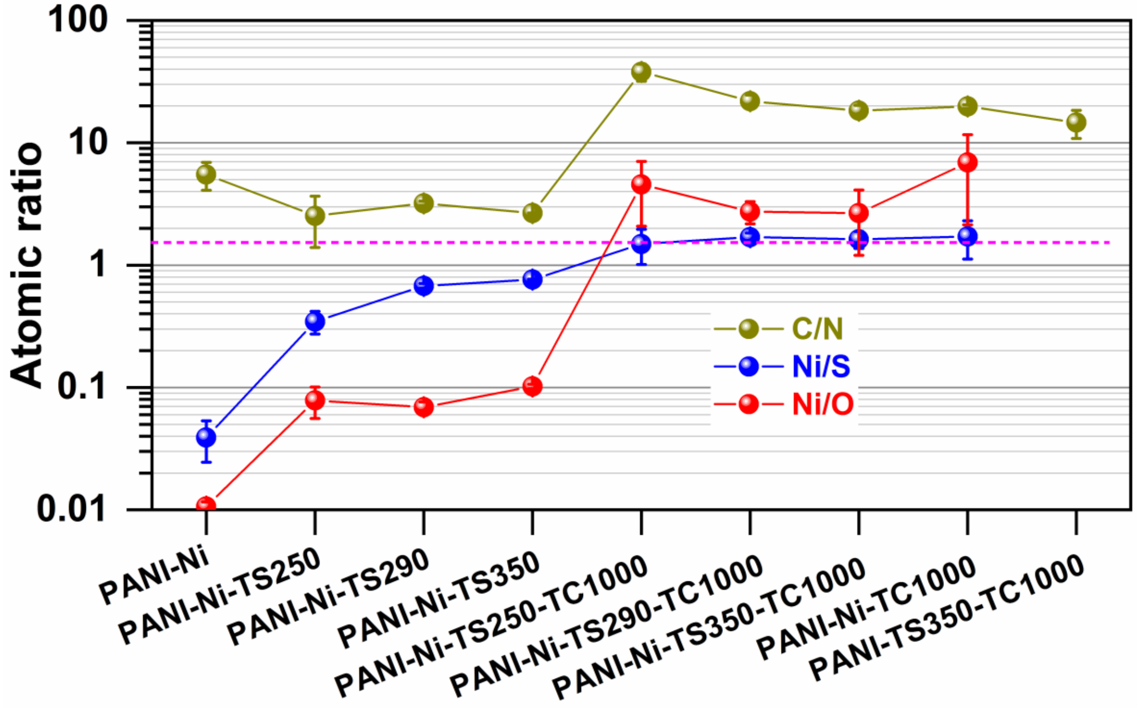

Supplementary Figure S9| Effect of the Thermal Stabilization: Physico-chemical characterization. Synthesis with 0.023 M Ni(+II). Results from EDX analysis. The dashed line represents the theoretical atomic ratio Ni/S = 1.5. Error bars represent one standard deviation (*n* ≥ 3)

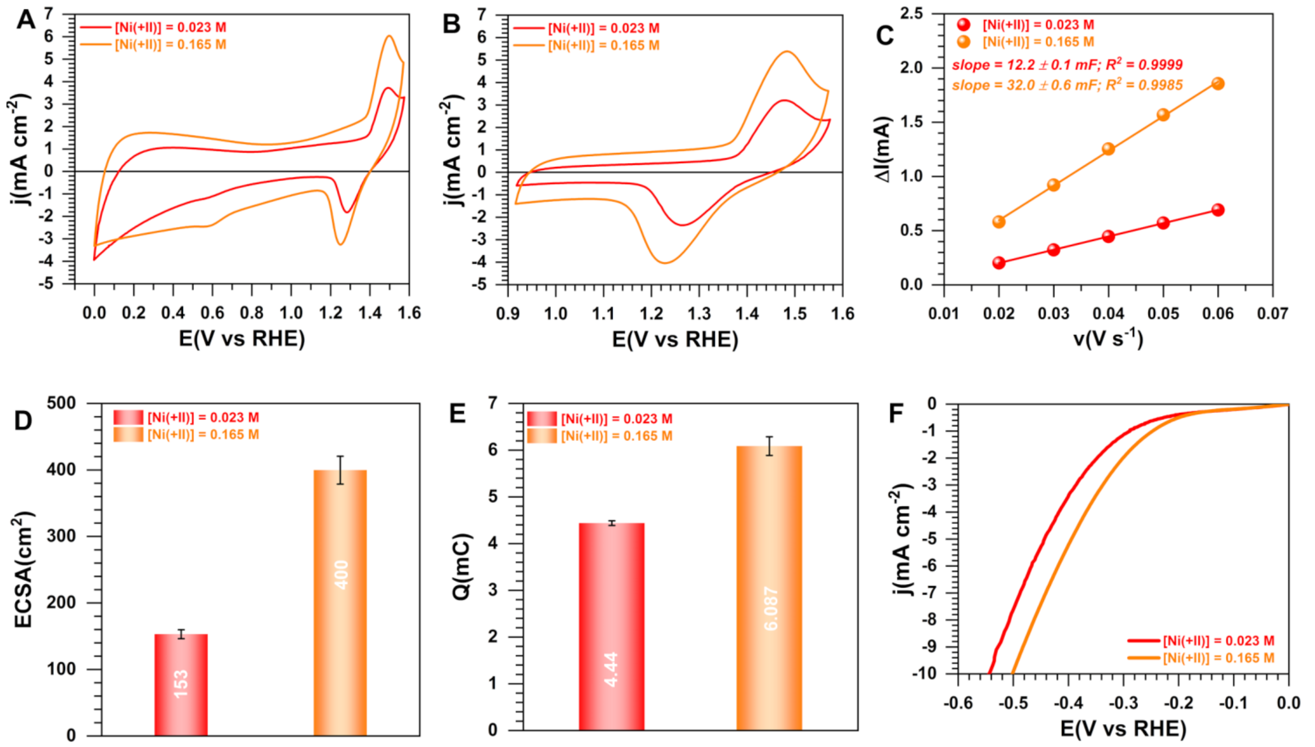

Supplementary Figure S10| Effect of the Ni(+II) concentration: Electrochemistry in 0.1 M KOH at 25 °C. (A) iR-uncorrected steady-state CVs recorded at 50 mV s^{-1} in a large potential window. **(B)** iR-uncorrected steady-state CVs recorded at 50 mV s^{-1} in a reduced potential window. **(C)** Plots of the absolute value of the charging current ($\Delta I = I_a - I_c$) of the double-layer region at 0.93 V vs RHE as a function of scan rate for determining the electrochemically active surface area (ECSA). **(D)** ECSA. **(E)** Exchange electrical charge (Q) of the faradaic process of NiO(OH) reduction peak. **(F)** iR-uncorrected HER polarization curves recorded at 5 mV s^{-1}. The investigated material is PANI-Ni-TS350-TC1000-2h. Working electrode is 1 cm^2 geometric surface area. Error bars represent one standard deviation ($n \geq 3$).

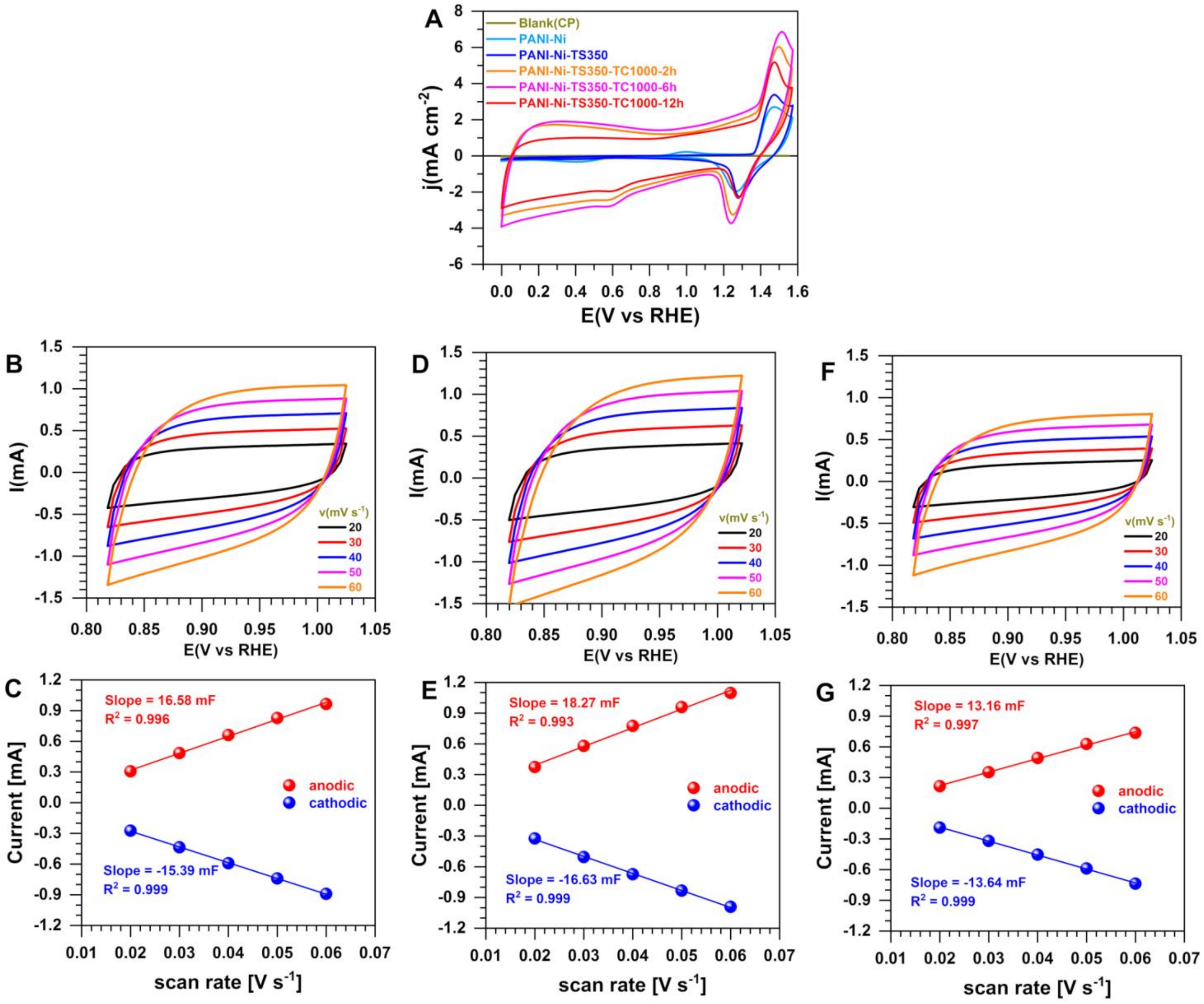

Supplementary Figure S11| Effect of the duration of the calcination (TC): Electrochemistry in 0.1 M KOH at 25 °C. Synthesis with 0.165 M Ni(+II). (A) iR-uncorrected steady-state CVs recorded at 50 mV s^{-1} in a large potential window. **(B-G)** Double-layer capacitance measurements for determining electrochemically active surface area (ECSA): **(B, D, F)** iR-uncorrected CVs recorded at different scan rates in the double-layer capacitance region and **(C, E, G)** The anodic (I_a) and cathodic (I_c) charging currents measured at 0.93 V vs RHE plotted as a function of scan rate; **(B, C)** PANI-Ni-TS350-TC1000-2h, **(D, E)** PANI-Ni-TS350-TC1000-6h, **(F, G)** PANI-Ni-TS350-TC1000-12h.

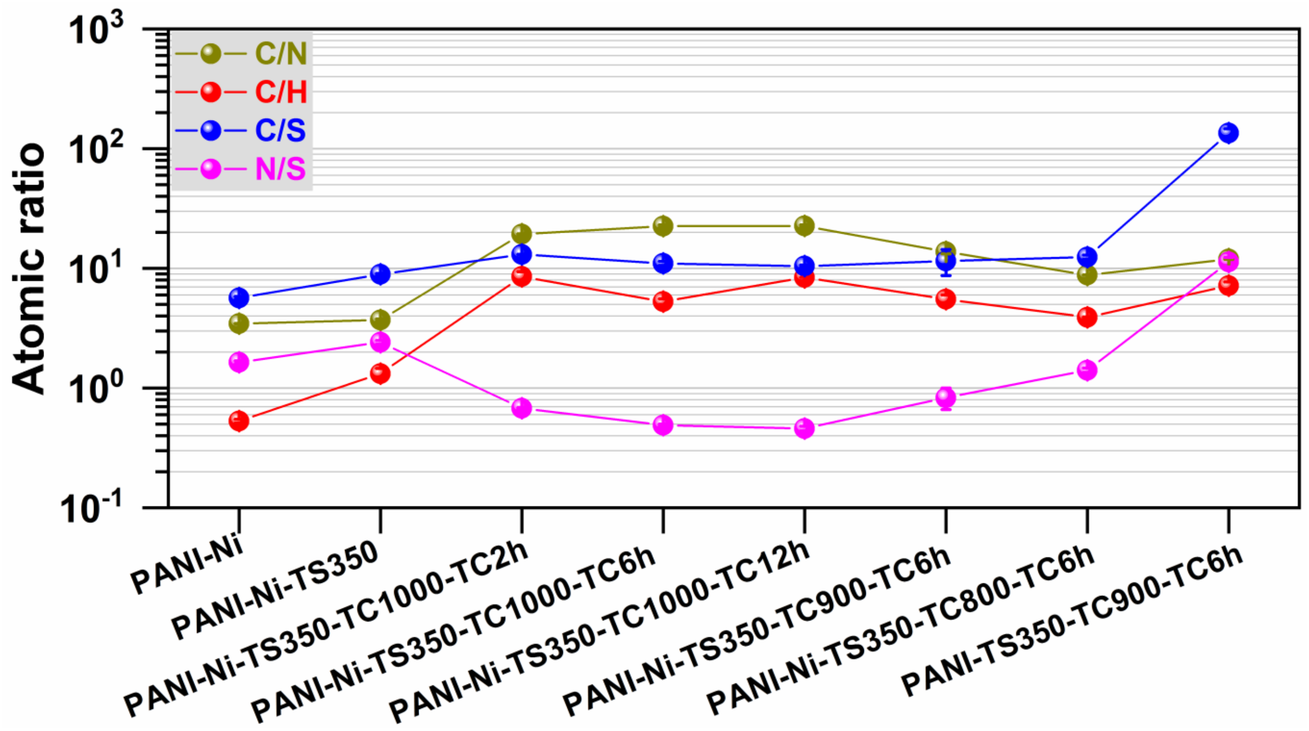

Supplementary Figure S12| Effect of the duration of the calcination (TC): Physico-chemical characterization. Synthesis with 0.165 M Ni(+II). Results from CHNS analysis. Error bars represent one standard deviation ($n \geq 3$)

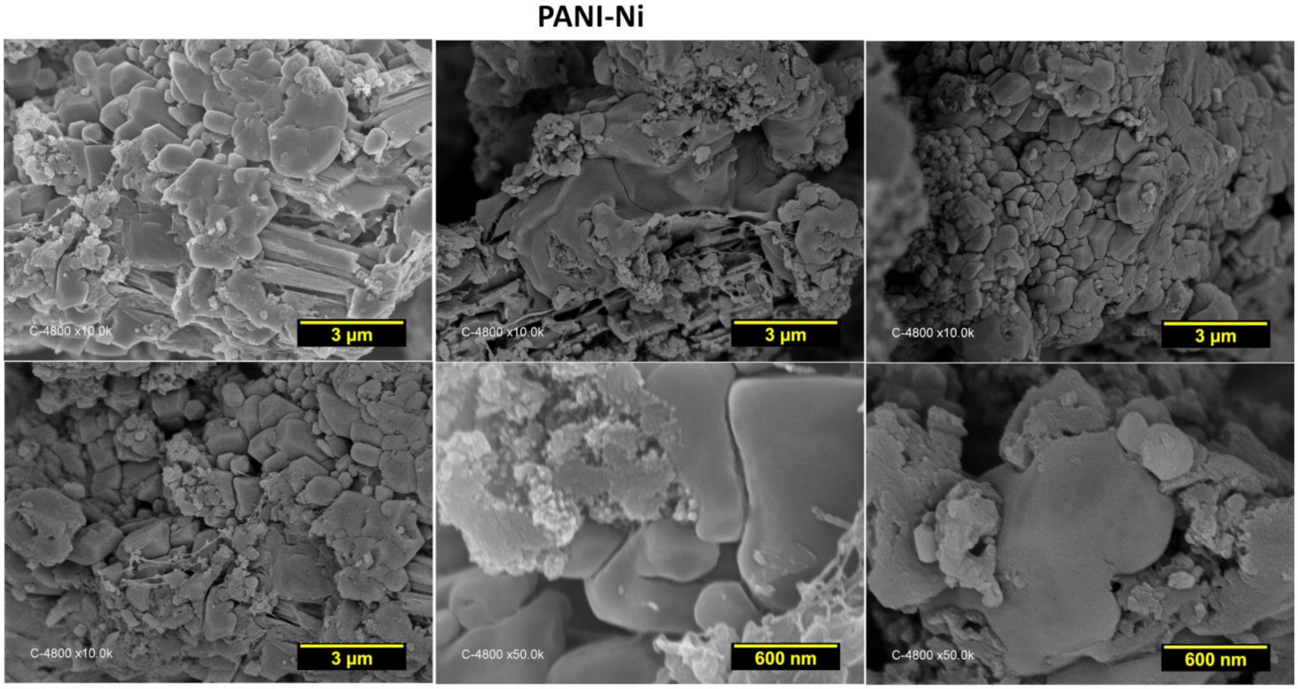

Supplementary Figure S13| Effect of the duration of the calcination (TC): Physico-chemical characterization. Synthesis with 0.165 M Ni(+II). SEM images of PANI-Ni.

PANI-Ni-ST350

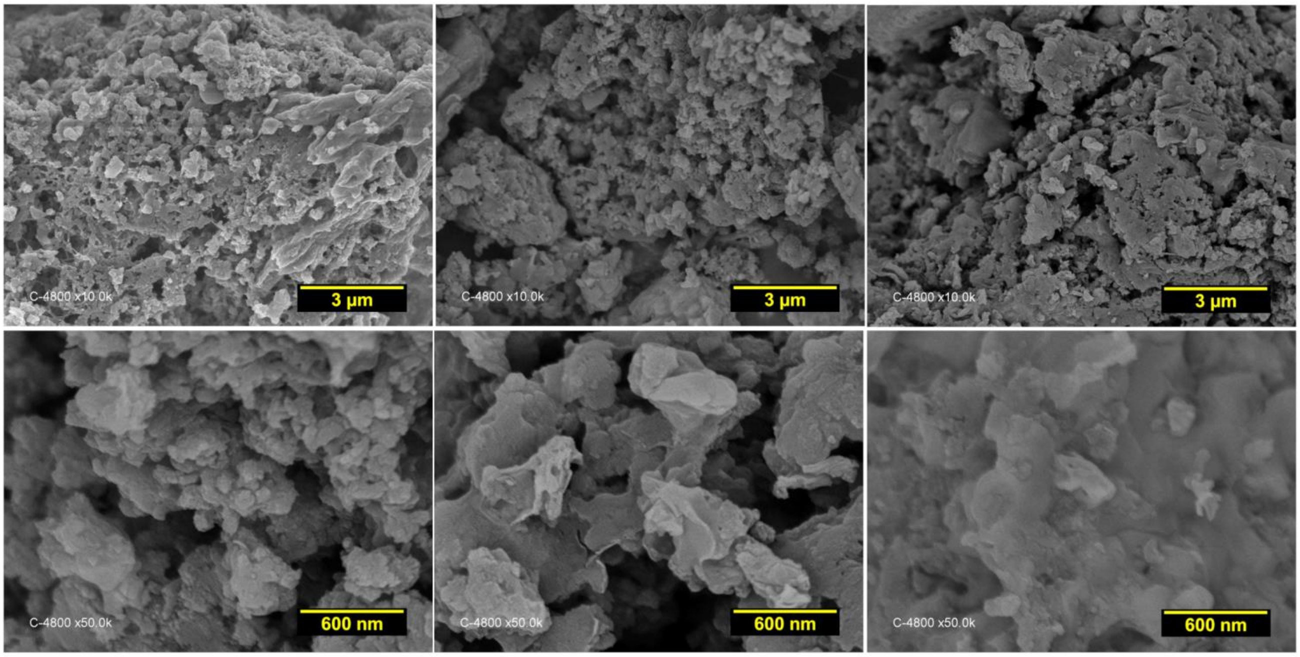

Supplementary Figure S14| Effect of the duration of the calcination (TC): Physico-chemical characterization. Synthesis with 0.165 M Ni(+II). SEM images of PANI-Ni-TS350.

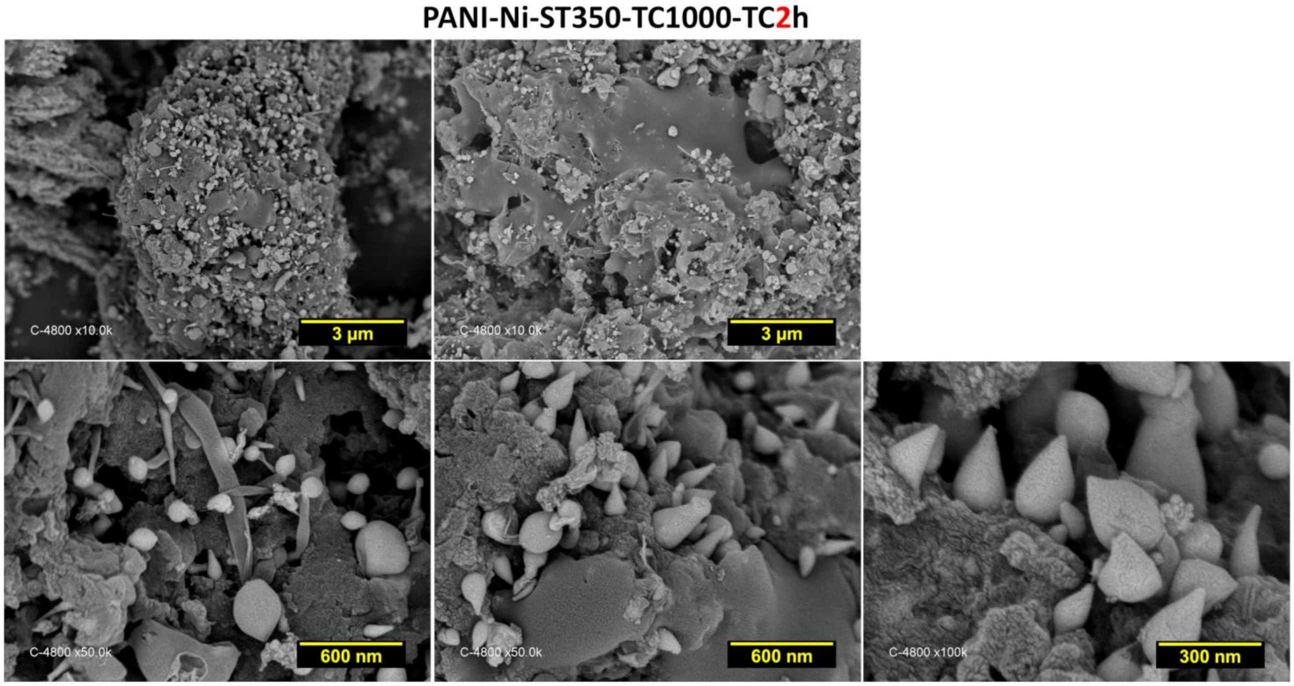

Supplementary Figure S15| Effect of the duration of the calcination (TC): Physico-chemical characterization. Synthesis with 0.165 M Ni(+II). SEM images of PANI-Ni-TS350-TC1000-TC2h.

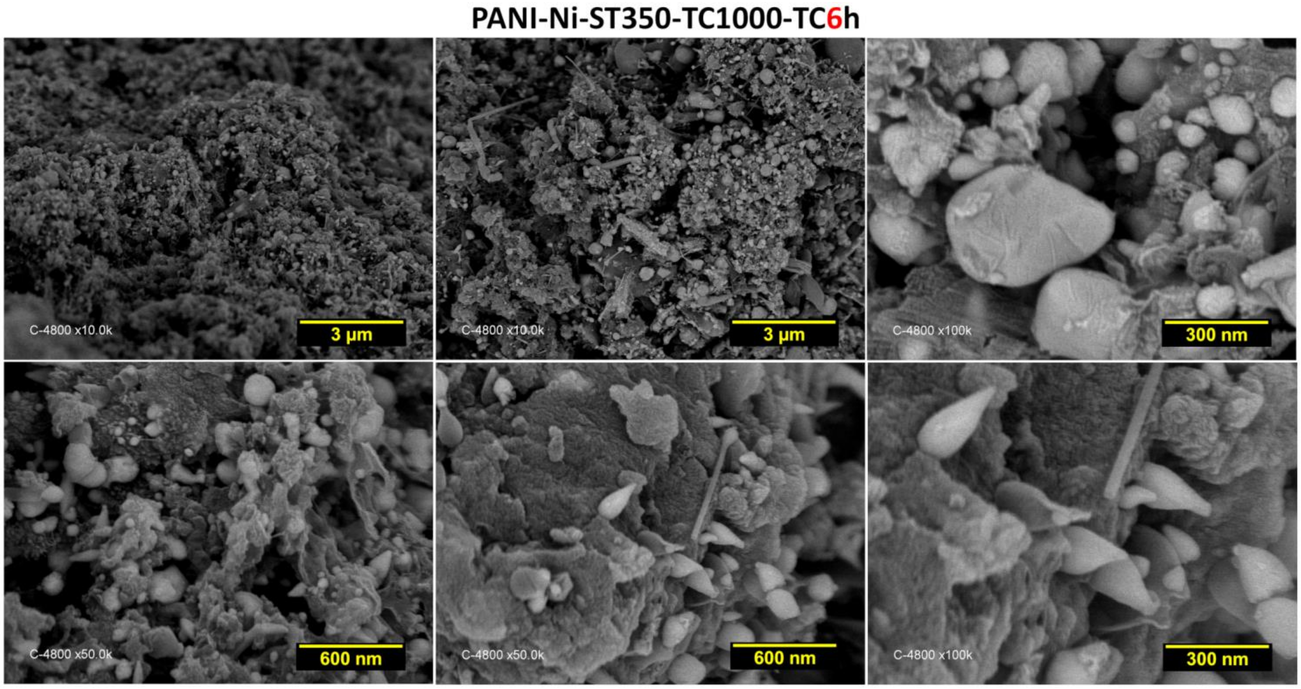

Supplementary Figure S16| Effect of the duration of the calcination (TC): Physico-chemical characterization. Synthesis with 0.165 M Ni(+II). SEM images of PANI-Ni-TS350-TC1000-TC6h.

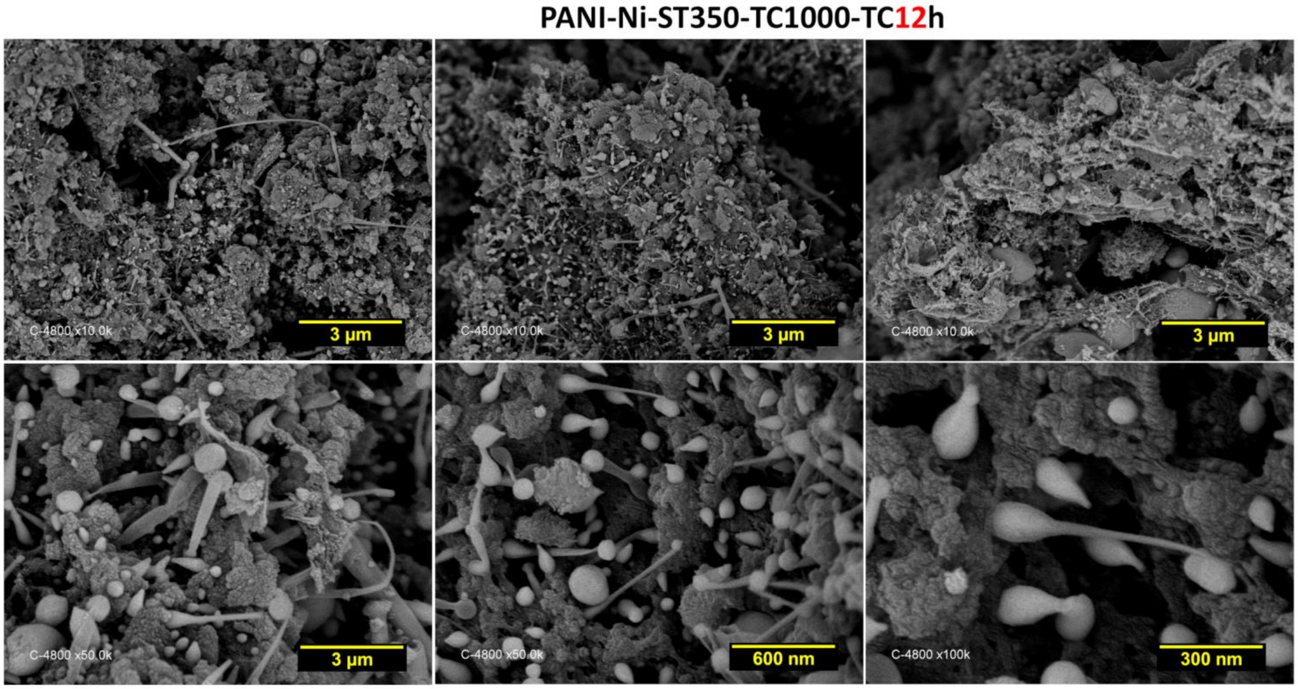

Supplementary Figure S17| Effect of the duration of the calcination (TC): Physico-chemical characterization. Synthesis with 0.165 M Ni(+II). SEM images of PANI-Ni-TS350-TC1000-TC12h.

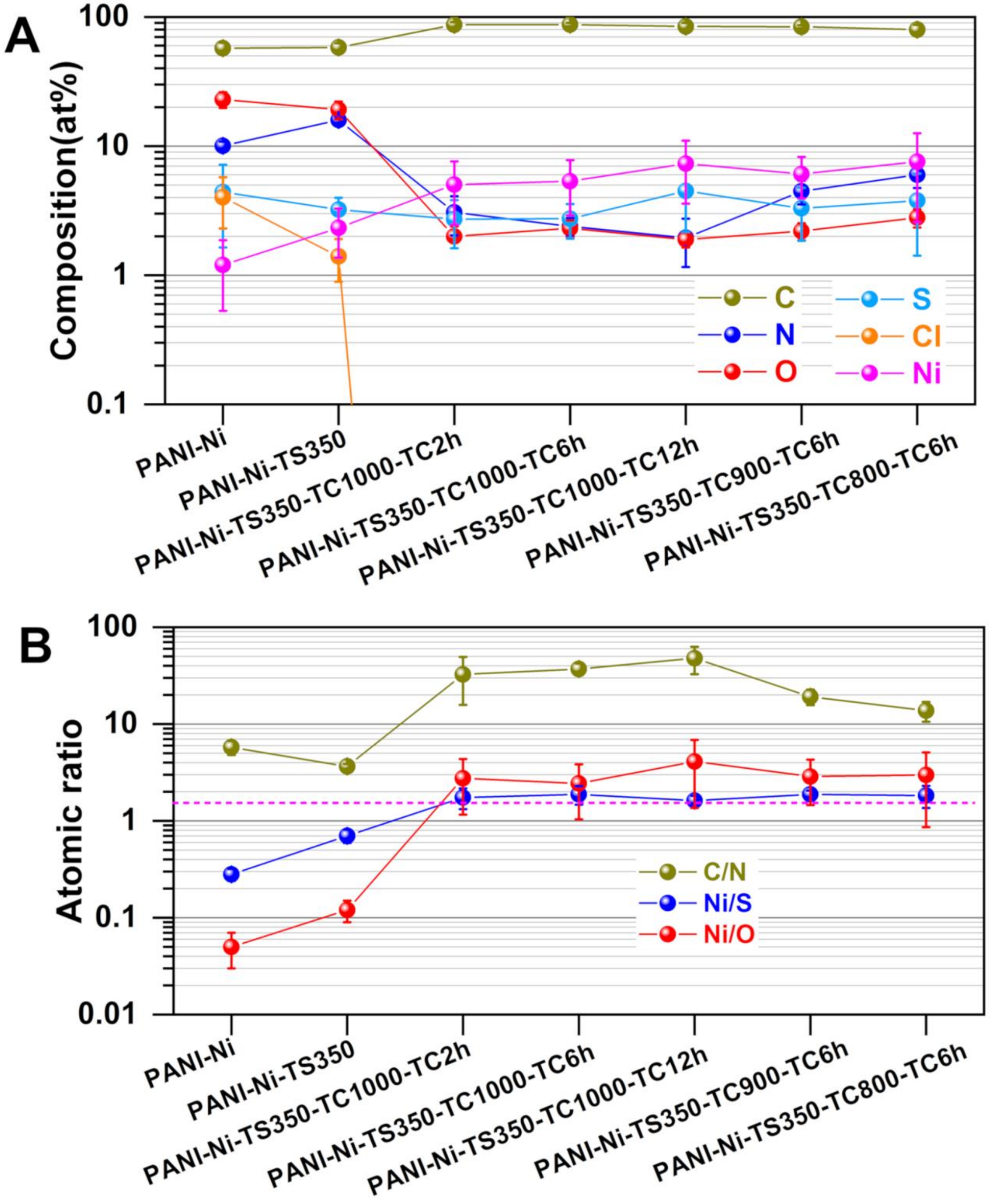

Supplementary Figure S18| Effect of the duration and temperature of the calcination (TC): Physico-chemical characterization. Synthesis with 0.165 M Ni(+II). Results from EDX analysis: **(A)** Atomic composition and **(B)** Atomic ratios. The dashed line represents the theoretical atomic ratio Ni/S = 1.5. Error bars represent one standard deviation ($n \geq 3$).

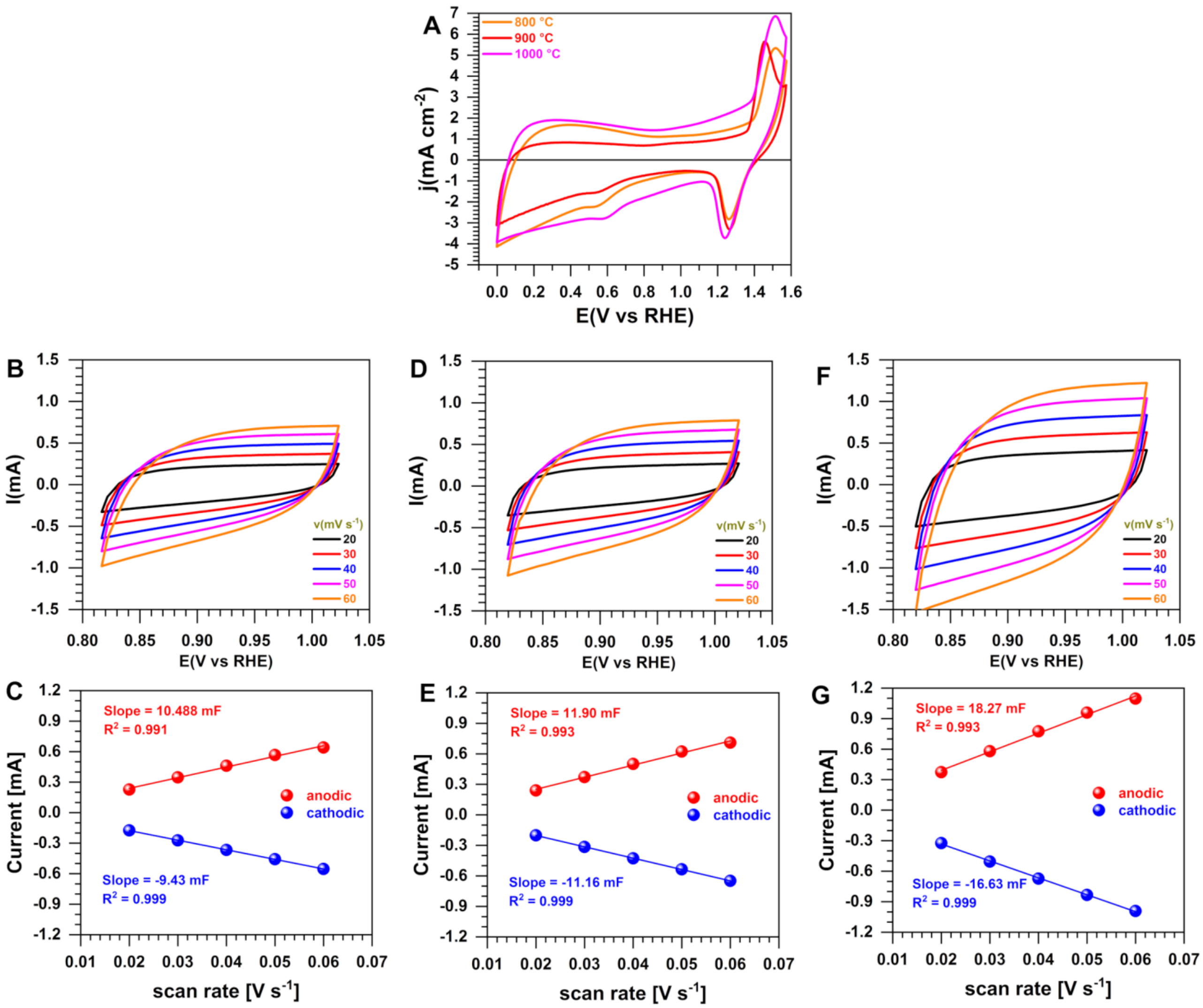

Supplementary Figure S19| Effect of the temperature of the calcination (TC): Electrochemistry in 0.1 M KOH at 25 °C. Synthesis with 0.165 M Ni(+II). (A) iR-uncorrected steady-state CVs recorded at 50 mV s^{-1} in a large potential window. **(B-G)** Double-layer capacitance measurements for determining electrochemically active surface area (ECSA): **(B, D, F)** iR-uncorrected CVs recorded at different scan rates in the double-layer capacitance region and **(C, E, G)** The anodic (I_a) and cathodic (I_c) charging currents measured at 0.93 V vs RHE plotted as a function of scan rate; **(B, C)** PANI-Ni-TS350-TC800, **(D, E)** PANI-Ni-TS350-TC900, **(F, G)** PANI-Ni-TS350-TC1000.

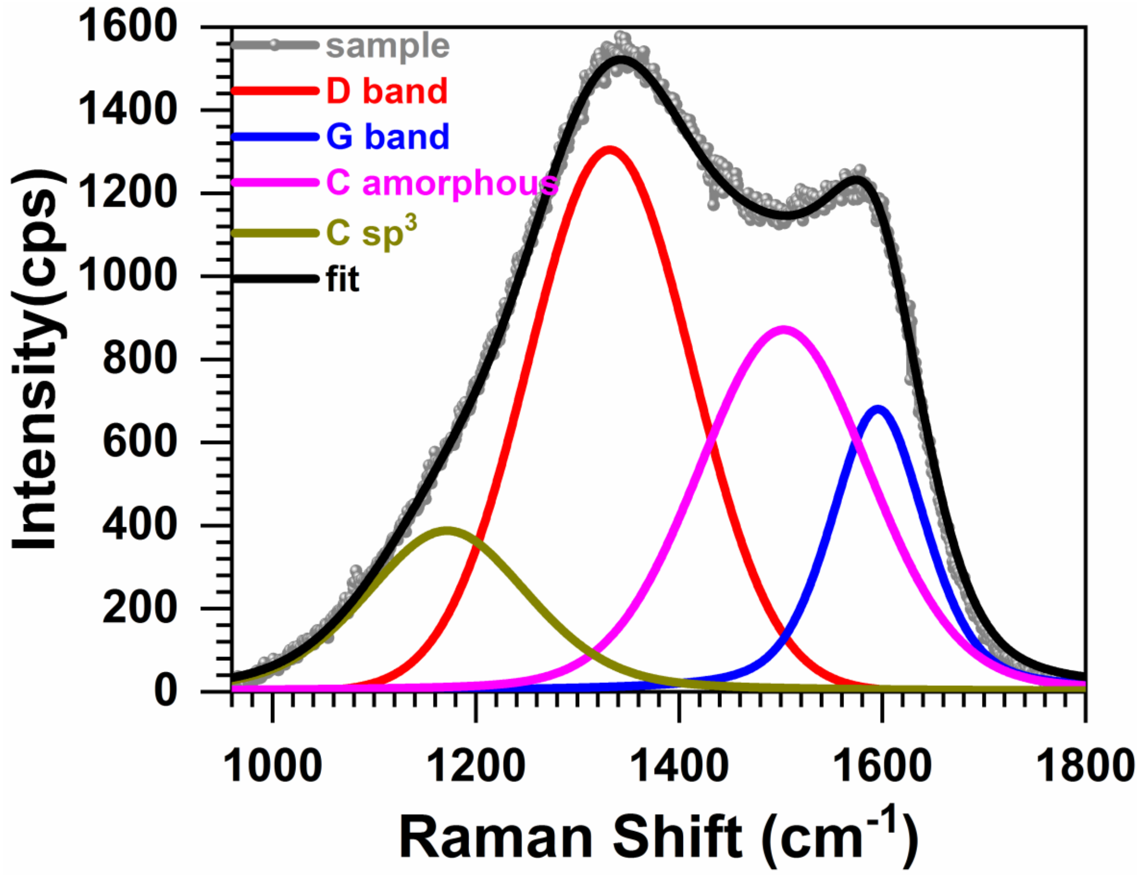

Supplementary Figure S20| Physico-chemical characterization. Raman spectroscopy of PANI-TS350-TC900-TC6h.

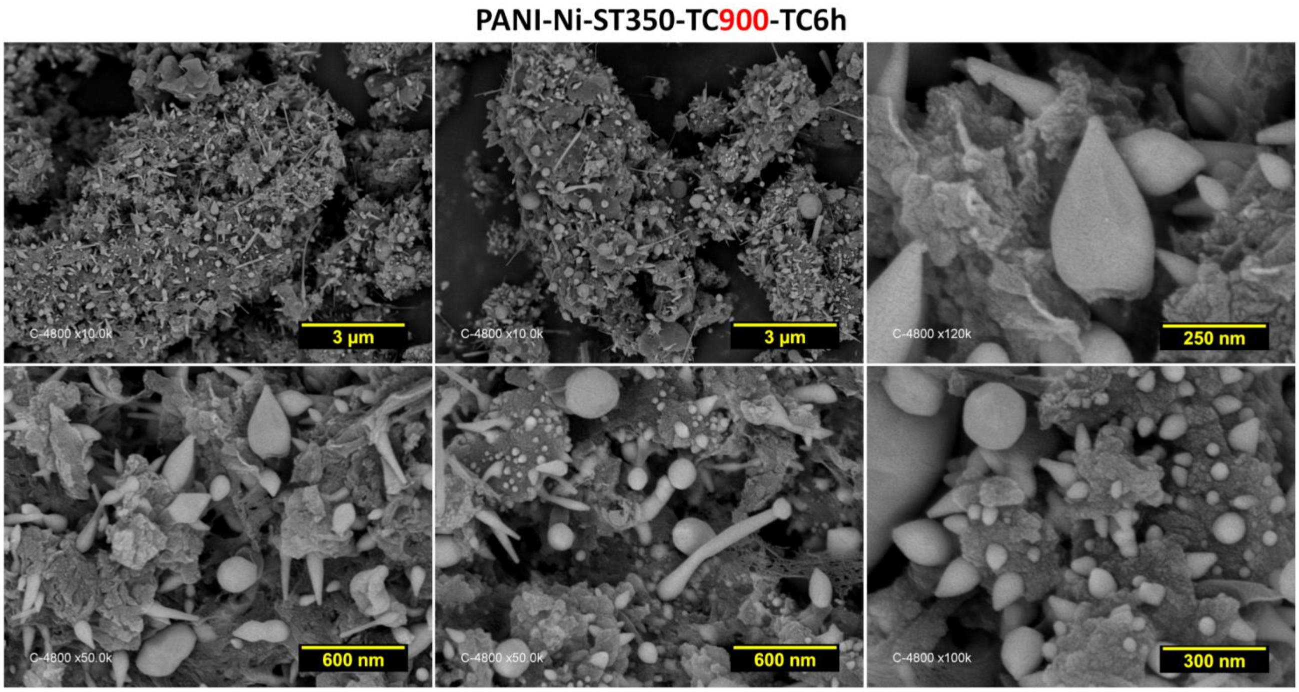

Supplementary Figure S21| Effect of the temperature of the calcination (TC): Physico-chemical characterization. Synthesis with 0.165 M Ni(+II). SEM images of PANI-Ni-TS350-TC900-TC6h.

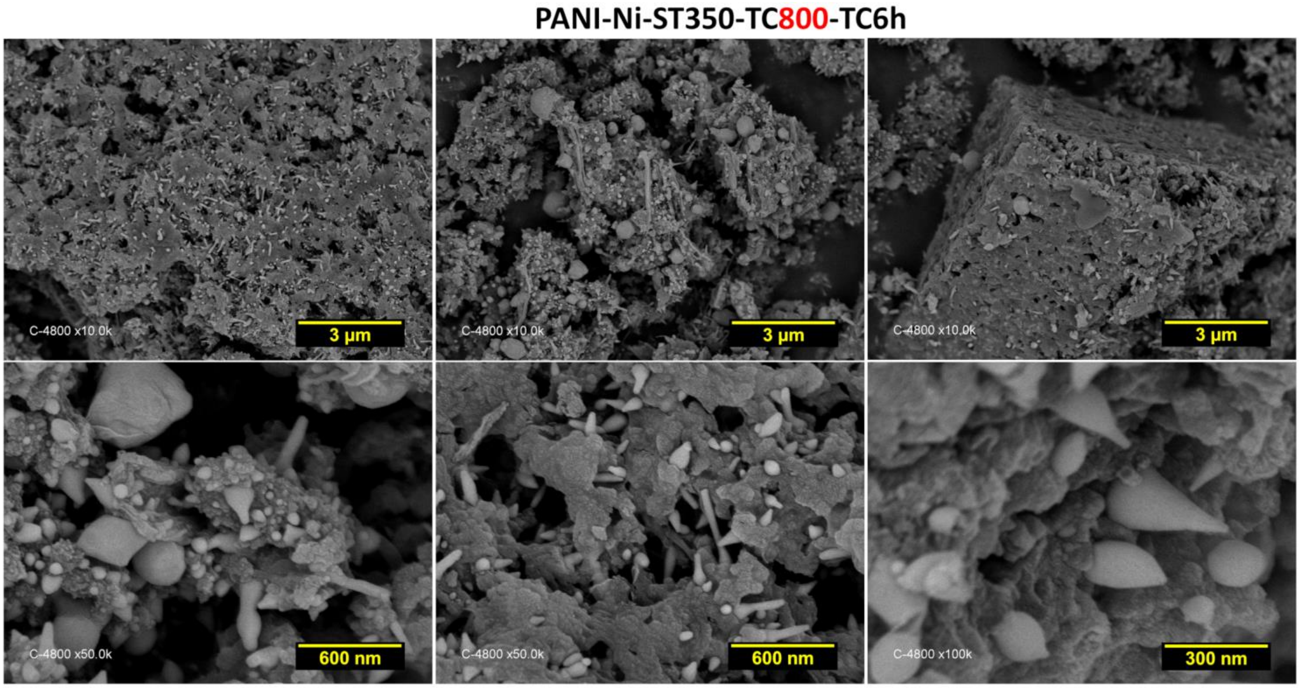

Supplementary Figure S22| Effect of the temperature of the calcination (TC): Physico-chemical characterization. Synthesis with 0.165 M Ni(+II). SEM images of PANI-Ni-TS350-TC800-TC6h.

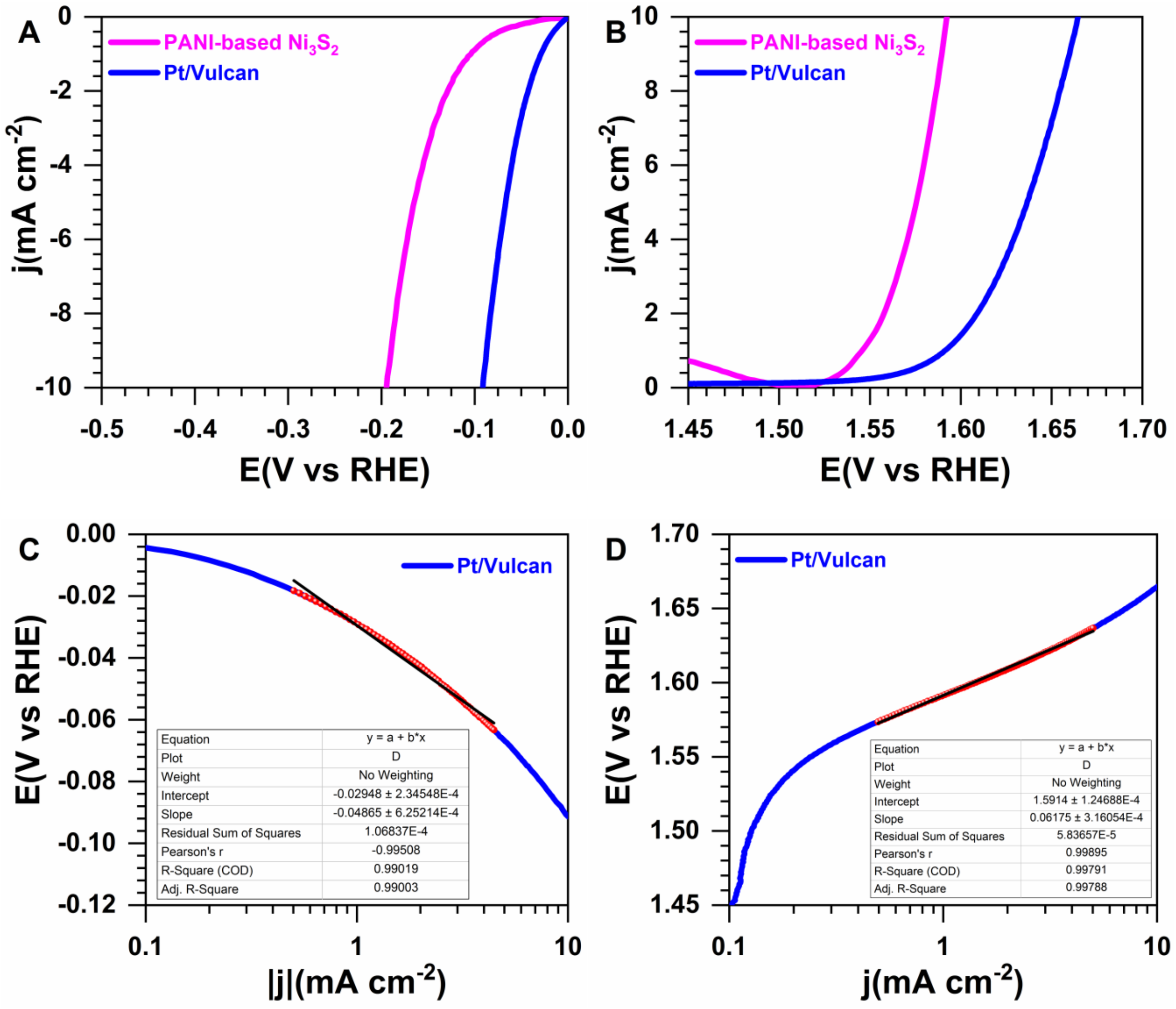

Supplementary Figure S23| Performance of commercial Pt/Vulcan and the as-synthesis PANI-based Ni_3S_2 with 0.165 M Ni(+II). (A) iR-free HER polarization curves recorded at 5 mV s^{-1}. **(B)** iR-free OER polarization curves recorded at 5 mV s^{-1}. **(C)** Tafel plots of HER by the current density. **(D)** Tafel plots of OER by the current density. Working electrode is 1 cm^2 geometric surface area and experiments were performed in 1 M KOH at 25 °C.

Comments: The catalytic ink for the commercial catalyst Pt/C (20 wt%, 2 nm, Premetek Co., USA) was prepared by ultrasonically mixing of 360 µL of isopropanol, 40 µL of Nafion® suspension and about 1 mg of Pt/C powder. Then, about 160 µL of the homogeneous ink was drop-casted onto each face of a bare L-shape CP electrode of 1 cm high and 0.5 cm width and dried at room temperature. This leads to a loading of about 0.08 mg_{Pt} cm^{-2}.

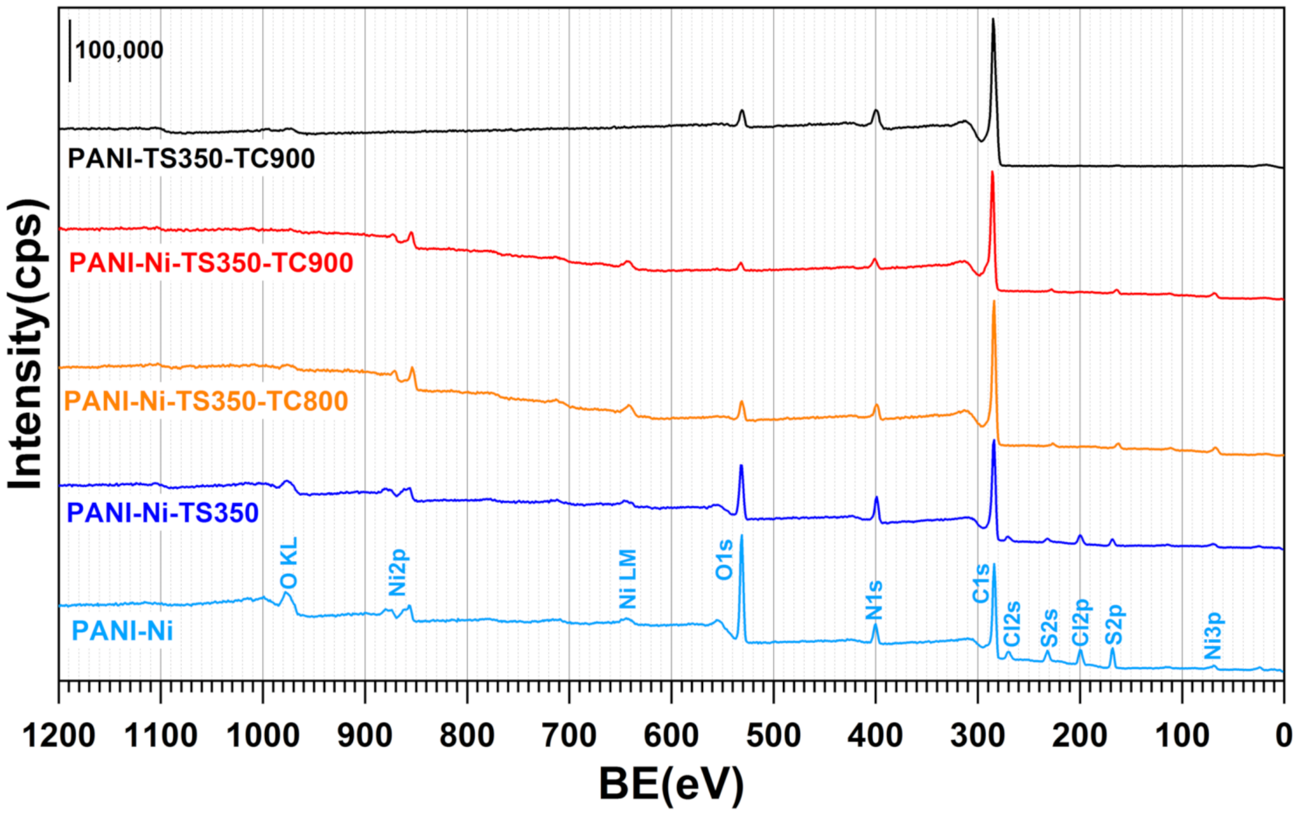

Supplementary Figure S24| Synthesis with 0.165 M Ni(+II): Compositional and structural characterization by XPS. Indexed survey (low resolution) XPS spectrum of the as-synthesized materials. Acquisition parameters: Pass energy = 150.0 eV; Energy step = 1.0 eV. The measurement of binding energy was corrected on the basis of the energy of C=C component of C1s at 284.4 eV.

Comments:

(1) One can see that the signal of Cl 2p diminishes after thermal stabilization (TS) and disappear completely after thermal calcination (TC).

(2) There no signal of Ni 2p for the sample PANI-TS350-TC900, which is normal since it is the blank material.

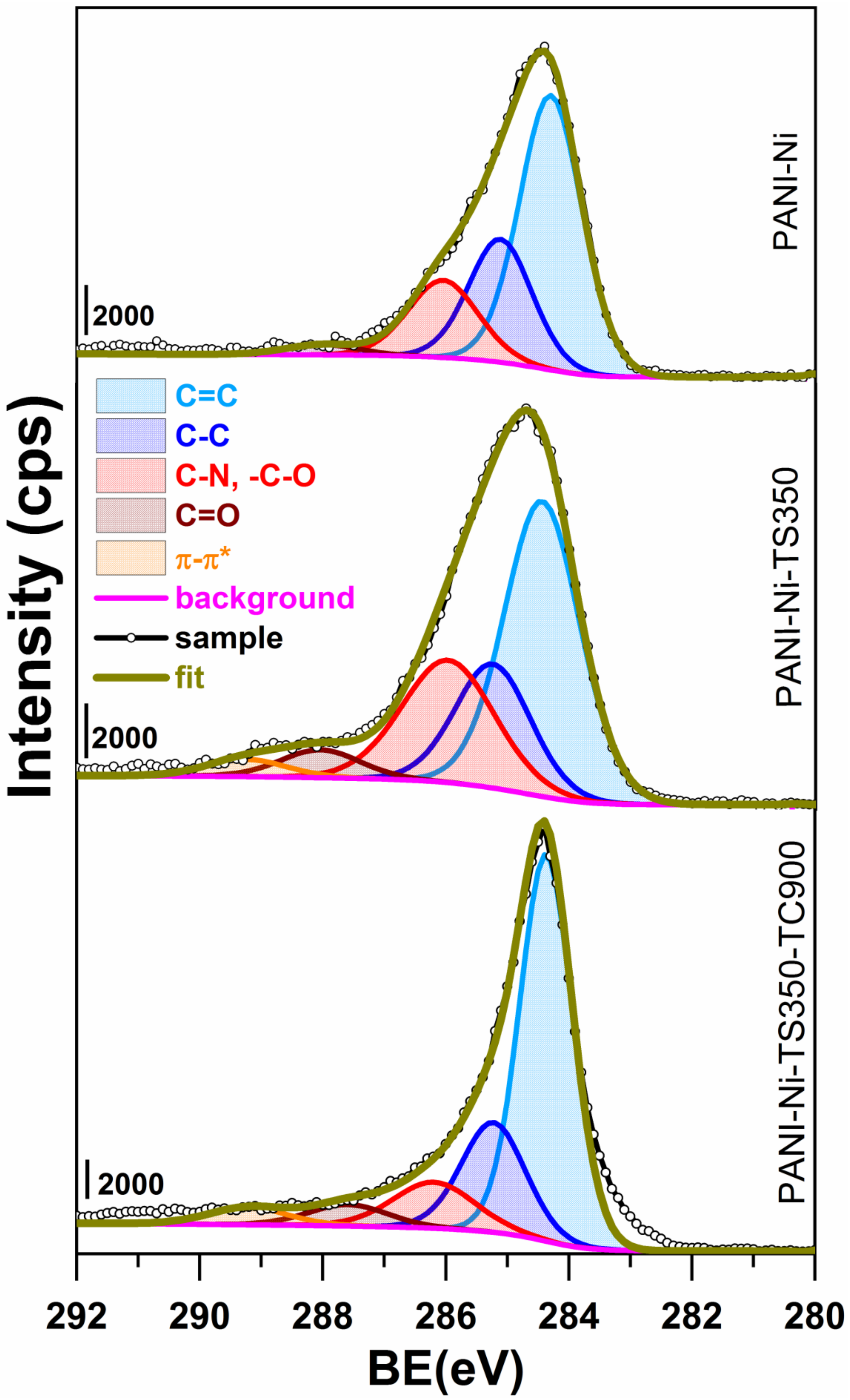

Intensity (cps)
PANI-Ni
PANI-Ni-TS350
PANI-Ni-TS350-TC900
2000
2000
2000
C=C
C-C
C-N, -C-O
C=O
π-π*
background
sample
fit
292
290
288
286
284
282
280
BE(eV)

Supplementary Figure S25| Synthesis with 0.165 M Ni(+II): Compositional and structural characterization by XPS. High-resolution spectra of C 1s for: **(A)** PANI-Ni, **(B)** PANi-Ni-TS350, and **(C)** PANI-Ni-TS350-TC900.

Comments: Based on the data available in the literature (Yu et al., 1990; Jiang et al., 2016), the compounds NiS, Ni_3S_2 and NiS_2 cannot really be differentiated in XPS. Also, given the size of the particles (up to hundreds of nanometers in some case), the small thickness of the analyzed (10 nm) does not allow distinguishing those compositions.

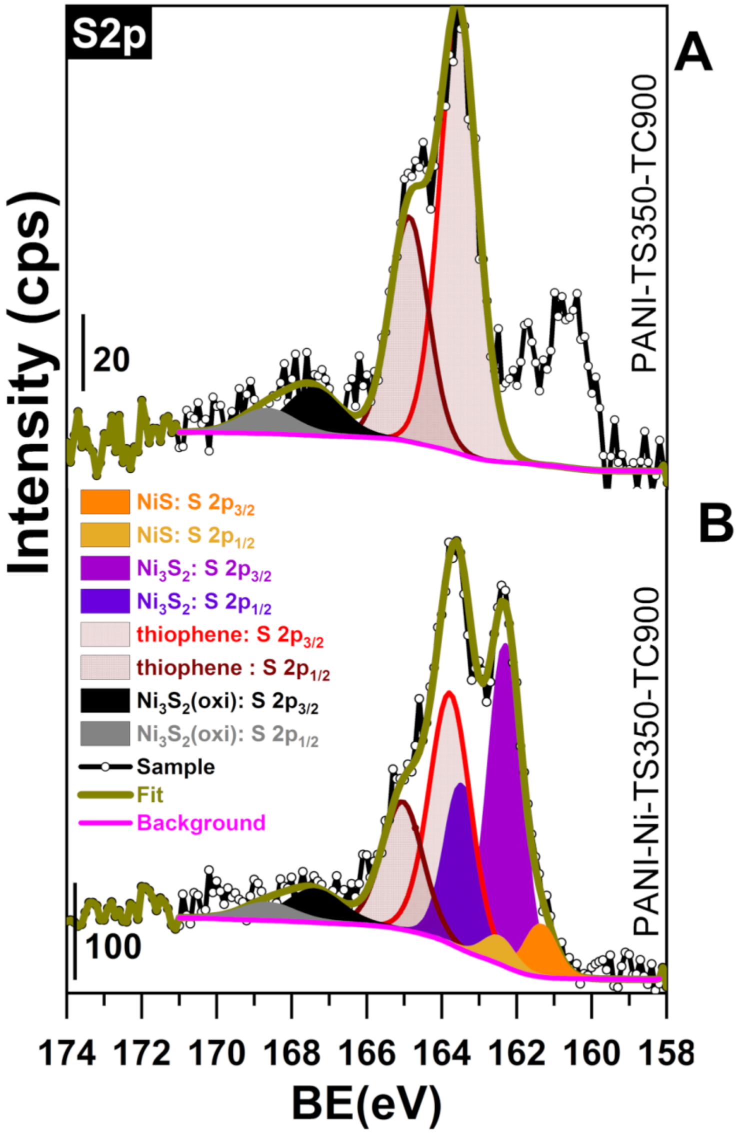

Supplementary Figure S26| Synthesis with 0.165 M Ni(+II): Compositional and structural characterization by XPS. Overlay high-resolution spectra of the S 2p for: **(A)** PANI- TS350-TC900, and **(B)** PANI-Ni-TS350-TC900.

Comments: The shoulder below 161 eV (very important for C 1s in the sample PANI-ST350-TC900, see below in **Figure S27**) is due to a charging effect (not enough charge compensation). It was not take into account during the fitting and quantification.

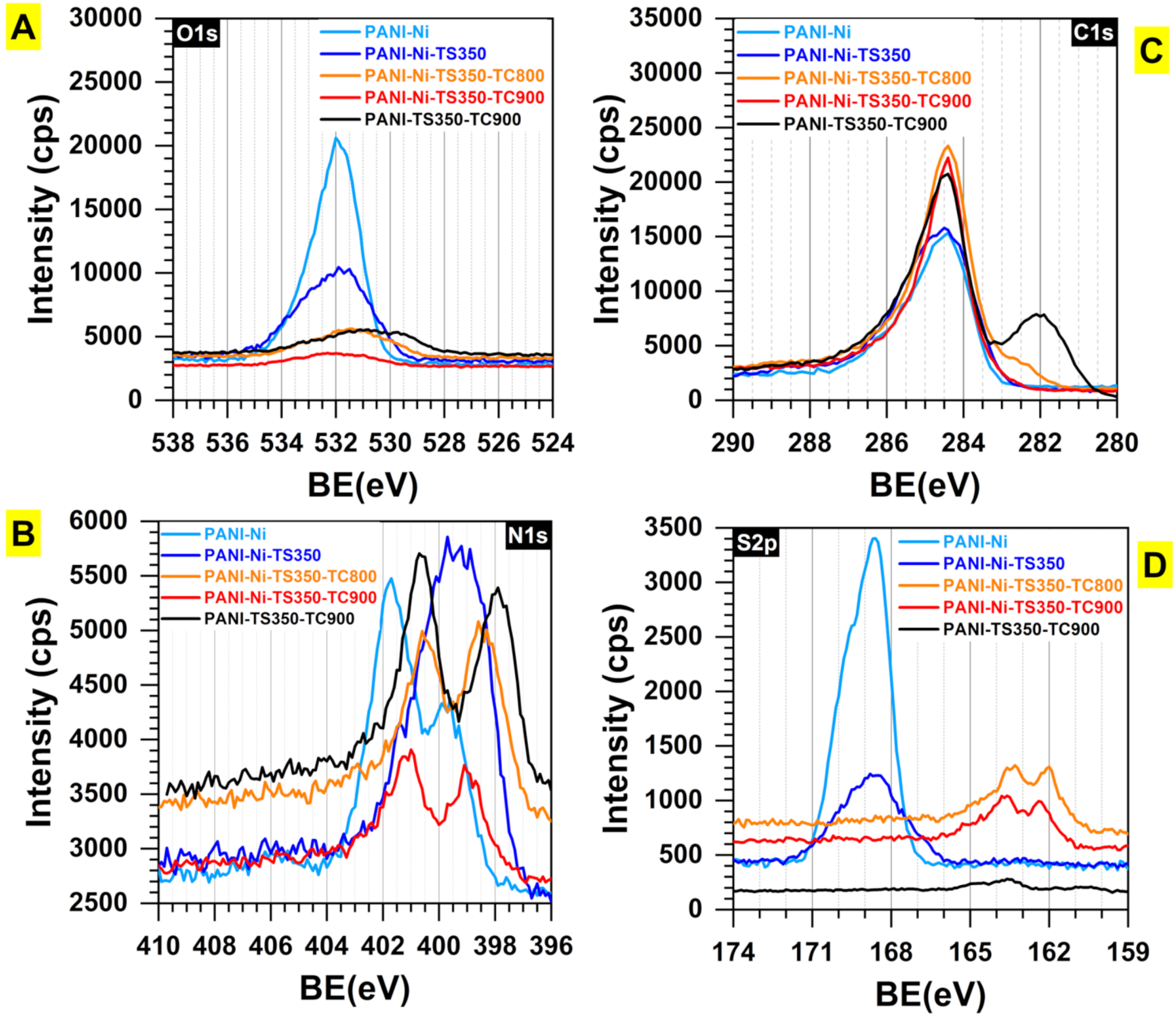

Supplementary Figure S27| Synthesis with 0.165 M Ni(+II): Compositional and structural characterization by XPS. Overlay high-resolution spectra of PANI-Ni, PANi-Ni-TS350, PANI-Ni-TS350-TC800, PANI-Ni-TS350-TC900, and PANI-TS350-TC900. **(A)** O 1s, **(B)** N 1s, **(C)** C 1s, and **(D)** S 2p.

<u>Comments</u>: There is a shoulder around 282 eV for C 1s, very important for the sample PANI-ST350-TC900, which is due to a charging effect (not enough charge compensation). It was not take into account during the fitting and quantification.

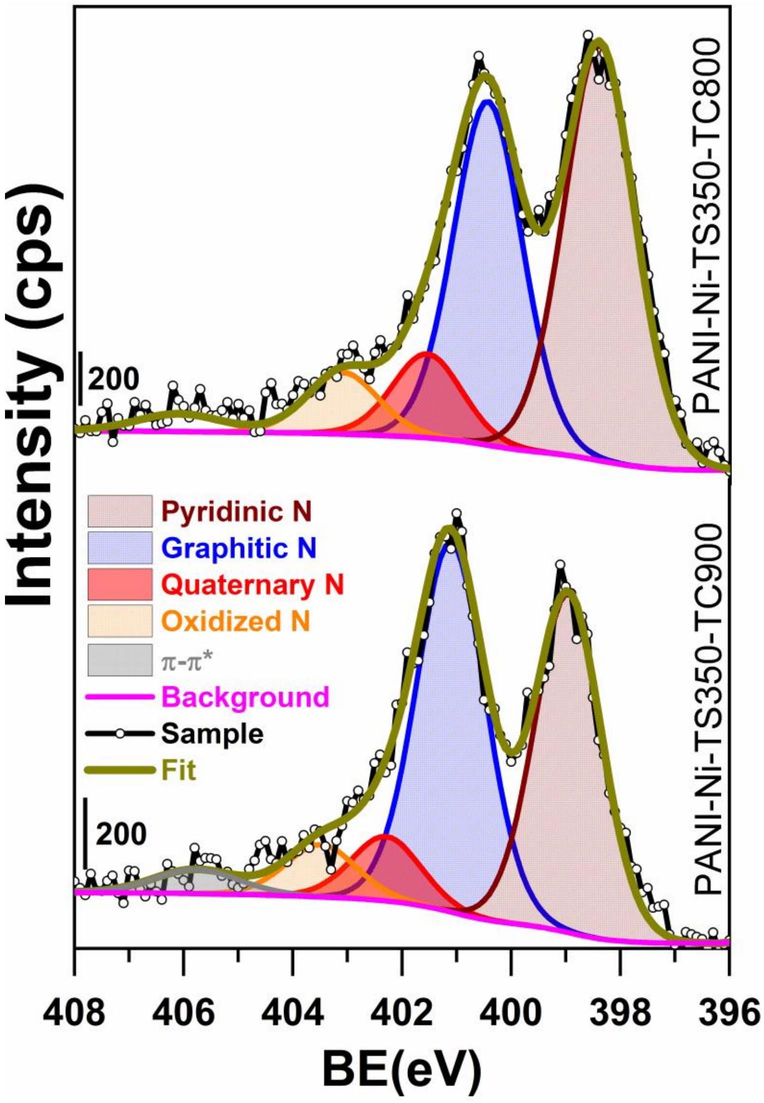

Supplementary Figure S28| Synthesis with 0.165 M Ni(+II): Compositional and structural characterization by XPS. High-resolution spectra of N 1s: **(A)** PANI-Ni-TS350-TC800 and **(B)** PANI-Ni-TS350-TC900

<u>Comments</u>: peak centered at 406 eV for π-π* transition was not take into account during the atomic distribution shown in **Figure S29**.

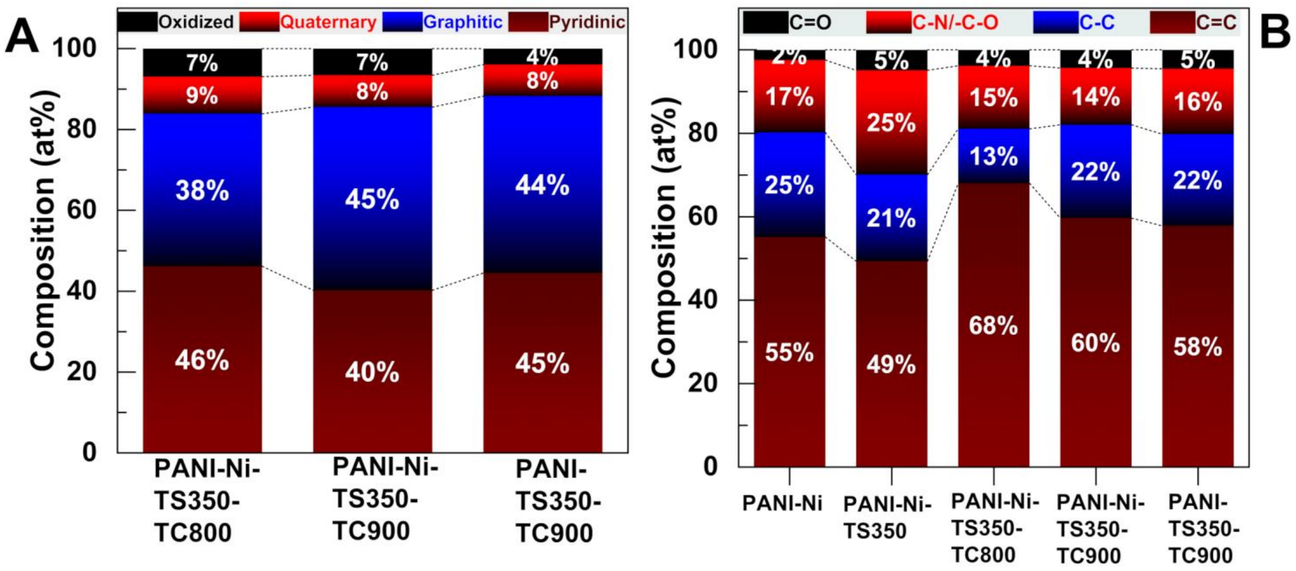

Supplementary Figure S29| Synthesis with 0.165 M Ni(+II): Compositional and structural characterization by XPS. Overall surface atomic composition for: **(A)** N-based species and **(B)** C-based species.

Supplementary Table S1| Fitted EIS data from the used EEC (R_Ω+Q_{CPE}//R_{ct}) to fit the data. Experiments were performed at a temperature of 25 °C.

		KOH [mol L^{-1}]	E (V vs RHE)	R_Ω (Ω cm^2)	R_{ct} (Ω cm^2)	Q_{CPE} (μF s$^{(1-n)}$)	n
[Ni(+II)] = 23 mM	PANi-Ni-TS250-TC1000	0.1	**-0.28**	**14.9**	**220**	**33**	**0.81**
			-0.33	14.8	105	47	0.81
			-0.38	14.5	43	40	0.81
			-0.43	14.6	**24**	36	0.82
	PANi-Ni-TS290-TC1000		**-0.28**	**14.3**	**132**	**78**	**0.80**
			-0.33	14.4	53	86	0.83
			-0.38	14.3	28	91	0.84
			-0.43	14.2	**17**	104	0.85
	PANi-Ni-TS350-TC1000		**-0.28**	**13.3**	**104**		**0.76**
			-0.33	13.2	47	72	0.78
			-0.38	13,2	25	68	0.77
			-0.43	13.1	**15**	67	0.79
[Ni(+II)] = 165 mM	PANi-R		-0.64	15.7	395	0.18	0.82
			-0.69	15.6	238	0.26	0.87
			-0.74	15.6	142	0.27	0.87
			-0.79	15.6	91	0.30	0.86
[Ni(+II)] = 0 mM	PANi-TS350-TC1000		-0.54	13.8	44	21	0.52
			-0.59	13.9	26	12	0.52
			-0.64	13.8	17	20	0.53
			-0.69	13,2	12	20	0.56
[Ni(+II)] = 165 mM	PANi-Ni-TS350-TC900		**-0.28**	**9.9**	**29**	**98**	**0.72**
			-0.33	9.8	20	72	0.72
			-0.38	9	12	67	0.71
			-0.43	9.9	9	56	0.72
	PANi-Ni-TS350-TC900 (before HER's ADT)	1	**-0.19**	**3.0**	**56**	**79**	**0.83**
			-0.24	3.0	19	84	0.87
			-0.29	3.1	12	78	0.83
			-0.34	3.0	6	69	0.83
	PANi-Ni-TS350-TC900 (after HER's ADT)		-0.14	3.1	79	80	0.88
			**-0.19**	**3.3**	**24**	**84**	**0.85**
			-0.24	3.3	10	71	0.85
			-0.29	3.3	6	64	0.82
	PANi-Ni-TS350-TC900 (before OER's ADT)		**1.51**	**3.6**	**102**	**41**	**0.81**
			1.56	3.5	32	29	0.80
			1.61	3.4	8	19	0.79
			1.69	3.3	3	12	0.76
	PANi-Ni-TS350-TC900 (after OER's ADT)		**1.51**	**3.0**	**72**	**64**	**0.82**
			1.56	3.1	10	31	0.74
			1.61	2.9	3	14	0.65
			1.69	2.8	2	1	0.58

Supplementary Table S2| Results from N_2 adsorption-desorption isotherms (77 K).

Entry		S_{BET} (m^2 g^{-1})	R_{BJH} (nm)	V_{tot} (cm^3 g^{-1})	V_{meso} (cm^3 g^{-1})	V_{micro} (cm^3 g^{-1})
[Ni(+II)] = 23 mM	PANI-Ni	4	32	0.031	0.015	0.016
	PANI-Ni-TS290	4	52	0.030	0.009	0.021
	PANI-Ni-TS290-TC1000	302	12	0.182	0.113	0.069
	PANI-Ni-TC1000	24	27	0.146	0.082	0.064
[Ni(+II)] = 0 mM	PANI-TS290-TC1000	65	27	0.162	0.090	0.072
[Ni(+II)] = 165 mM	PANI-Ni-TS350-TC1000-2h	190	16	0.170	0.145	0.025
	PANI-Ni-TS350-TC1000-6h	210	14	0.168	0.154	0.014
	PANI-Ni-TS350-TC1000-12h	215	13	0.154	0.153	0.001
	PANI-Ni-TS350-TC900-6h	195	22	0.165	0.133	0.032
	PANI-Ni-TS350-TC800-6h	216	15	0.153	0.149	0.004
[Ni(+II)] = 0 mM	PANI-TS350-TC900-6h	155	17	0.165	0.148	0.017

Supplementary Table S3| CHNS analysis of the materials synthesized under 0.023 M Ni(+II). Control synthesis in the absence of Ni (+II). Standard deviation is determined from *n* ≥ 3.

Entry		PANI-Ni-TS350-TC1000	PANI-Ni-TS290-TC1000	PANI-Ni-TC1000
Total mass (wt.%)	C	67.07±0.12	69.63±0.09	72.85±0.02
	H	0.95±0.03	1.29±0.02	1.15±0.01
	N	6.38±0.11	5.32±0.04	5.50±0.03
	S	5.19±0.32	4.71±0.16	4.30±0.02
	total	79.60±0.52	80.95±0.08	83.80±0.03
	balanced	20.40±0.52	19.05±0.08	16.20±0.03
Atomic ratio	C/N	12.26±0.19	15.27±0.13	15.46±0.08
	C/H	5.93±0.22	4.54±0.07	5.31±0.05
	C/S	34.54±2.06	39.49±1.36	45.25±0.19
	N/S	2.82±0.12	2.59±0.07	2.93±0.03

Supplementary Table S4| Results from O analysis. SD = Standard Deviation (*n* ≥ 3)

Entry		O(wt%)	
		Average	SD
[Ni(+II)] = 23 mM	PANI-Ni-TS350-TC1000	5.5	0.2
	PANI-Ni-TC1000	7.1	0.4
[Ni(+II)] = 165 mM	PANI-Ni-TS350-TC1000-2h	2.9	0.1
	PANI-Ni-TS350-TC1000-6h	4.7	0.5
[Ni(+II)] = 0 mM	PANI-TS350-TC900-6h	5.2	0.2

Supplementary Table S5| Results from ICP. %RSD = Relative Standard Deviation

Entry		Ni(wt%)	%RSD
[Ni(+II)] = 23 mM	PANI-Ni-TS350-TC1000-2h	6.9	0.52
[Ni(+II)] = 165 mM	PANI-Ni-TS350-TC900-6h	31.8	1.33

Supplementary Table S6| Results from the Raman spectroscopy. L_a: in-plane crystallite size. A_D/A_G: integrated area ratios of D to G. FWHM: full width at half-maximum.

Entry		D		G		A_D/A_G	L_a (nm)
		ν_G (cm^{-1})	FWHM (cm^{-1})	ν_G (cm^{-1})	FWHM (cm^{-1})		
[Ni(+II)] = 23 mM	PANI-Ni-TS250-TC1000-2h	1311	185	1589	100	2.9	15.8
	PANI-Ni-TS290-TC1000-2h	1318	180	1589	100	3.0	14.9
	PANI-Ni-TS350-TC1000-2h	1330	185	1594	100	3.1	14.6
	PANI-Ni-TC1000-2h	1329	195	1592	97	3.1	14.8
[Ni(+II)] = 0 mM	PANI-TS350-TC1000-2h	1331	230	1591	115	3.3	13.9
[Ni(+II)] = 165 mM	PANI-Ni-TS350-TC1000-2h	1348	215	1595	97	3.5	12.9
	PANI-Ni-TS350-TC1000-6h	1333	160	1588	87	2.1	21.7
	PANI-Ni-TS350-TC1000-12h	1323	180	1588	100	2.8	16.2
	PANI-Ni-TS350-TC900-6h	1335	180	1585	87	2.2	20.9
	PANI-Ni-TS350-TC800-6h	1325	170	1590	100	2.8	16.4
[Ni(+II)] = 0 mM	PANI-TS350-TC900-6h	1332	190	1596	110	2.9	15.5

Supplementary Table S7| CHNS analysis of the materials synthesized under 0.165 M Ni(+II). Control synthesis in the absence of Ni (+II). Standard deviation is determined from $n \geq 3$.

Entry		PANI- Ni	PANI-Ni-TS350	PANI-Ni-TS350-TC1000-TC2h	PANI-Ni-TS350-TC1000-TC6h	PANI-Ni-TS350-TC1000-TC12h	PANI-Ni-TS350-TC900-TC6h	PANI-Ni-TS350-TC800-TC6h	PANI-TS350-TC900-TC6h
Total mass (wt.%)	C	25.6±0.2	34.2±0.1	50.4±0.4	46.7±0.1	48.0±0.3	48.0±0.3	45.8±0.1	82.9±0.1
	H	4.1±0.1	2.2±0.2	0.5±0.1	0.7±0.1	0.5±0.1	0.7±0.1	1.0±0.1	1.0±0.1
	N	8.7±0.1	10.8±0.2	3.0±0.1	2.4±0.1	2.5±0.1	4.1±0.2	6.1±0.1	8.1±0.1
	S	12.1±0.3	10.2±0.2	10.3±0.1	11.4±0.4	12.3±0.2	11.5±2.9	9.9±0.4	1.6±0.1
	total	50.4±0.1	57.4±0.4	64.2±0.4	61.3±0.6	63.2±0.2	64.3±3.5	62.7±0.4	93.6±0.1
	balanced	49.6±0.1	42.6±0.4	35.8±0.4	38.7±0.6	36.8±0.2	35.7±3.5	37.3±0.5	6.4±0.1
Relative C+H+N +S (at.%)	C	29.8±0.5	46.6±1.7	80.3±0.8	75.5±0.9	79.4±0.1	74.4±2.2	68.9±0.2	81.2±0.7
	H	56.2±0.6	35.5±2.5	9.4±0.8	14.3±0.6	9.4±0.1	13.5±0.8	17.7±0.1	11.4±0.7
	N	8.7±0.1	12.6±0.7	4.2±0.1	3.3±0.1	3.5±0.1	5.4±0.1	7.8±0.1	6.8±0.1
	S	5.3±0.1	5.2±0.1	6.1±0.1	6.9±0.2	7.6±0.1	6.6±1.4	5.6±0.2	0.6±0.1
Atomic ratio	C/N	3.45±0.01	3.70±0.08	19.33±0.13	22.56±0.55	22.57±0.51	13.76±0.52	8.79±0.02	11.94±0.07
	C/H	0.53±0.02	1.32±0.14	8.55±0.83	5.27±0.30	8.41±0.01	5.52±0.47	3.90±0.03	7.17±0.52
	H/N	6.50±0.16	2.83±0.37	2.27±0.20	4.28±0.14	2.68±0.06	2.50±0.12	2.26±0.02	1.67±0.13
	C/S	5.67±0.17	8.91±0.16	13.07±0.04	11.00±0.43	10.45±0.20	11.52±2.84	12.41±0.49	135.05±11.49
	N/S	1.64±0.05	2.41±0.09	0.68±0.01	0.49±0.01	0.46±0.02	0.83±0.17	1.41±0.06	11.32±1.02

Supplementary Table S8| EDX analysis of the materials synthesized under 0.165 M Ni(+II). Standard deviation is determined from *n* ≥ 3.

Entry		PANI- Ni	PANI- Ni-TS350	PANI- Ni-TS350-TC1000-TC2h	PANI- Ni-TS350-TC1000-TC6h	PANI- Ni-TS350-TC1000-TC12h	PANI- Ni-TS350-TC900-TC6h	PANI- Ni-TS350-TC800-TC6h
Weight	C(wt%)	44.6±6.6	46.2±5.8	70.3±10.7	69.4±8.3	62.8±11.9	64.6±7.1	60.1±17.2
	N(wt%)	9.1±0.6	14.8±1.3	2.8±0.8	2.2±0.5	1.7±0.7	4.0±1.2	5.2±1.3
	O(wt%)	23.6±2.2	20.1±2.2	2.2±0.7	2.5±0.5	1.9±0.4	2.3±0.5	2.8±1.0
	S(wt%)	9.0±5.2	6.8±1.2	5.7±1.9	5.4±1.8	8.4±3.1	6.6±2.4	6.9±3.8
	Cl(wt%)	9.3±4.1	3.2±1.0	0	0	0	0	0
	Ni(wt%)	4.5±2.3	8.9±3.1	19.0±8.9	20.2±8.0	25.2±9.5	22.5±6.3	25.0±15.2
Atomic	C(at%)	57.34±5.99	58.01±4.56	87.17±4.11	87.22±2.71	84.38±5.74	83.95±2.38	79.84±7.11
	N(at%)	10.05±0.79	15.90±0.81	3.07±1.03	2.39±0.38	1.95±0.79	4.47±0.93	6.00±1.26
	O(at%)	22.97±3.21	19.15±3.03	2.00±0.38	2.31±0.35	1.89±0.25	2.20±0.33	2.79±0.45
	S(at%)	4.41±2.77	3.22±0.76	2.72±1.10	2.74±0.82	4.49±2.32	3.30±1.45	3.78±2.36
	Cl(at%)	4.02±1.72	1.40±0.51	0	0	0	0	0
	Ni(at%)	1.20±0.67	2.33±0.96	5.04±2.58	5.34±2.43	7.31±3.72	6.09±2.17	7.58±5.01
Atomic ratio	C/N	5.75±0.95	3.65±0.22	32.56±16.78	36.98±5.24	47.72±15.03	19.14±3.47	13.74±3.18
	Ni/S	0.28±0.04	0.70±0.11	1.74±0.42	1.88±0.41	1.62±0.19	1.88±0.17	1.83±0.47
	Ni/O	0.05±0.02	0.12±0.03	2.75±1.59	2.44±1.40	4.11±2.75	2.87±1.41	2.98±2.12

Supplementary Table S9| Comparative performance of relevant HER on Ni-based catalysts from literature in alkaline solution. CNNs = carbon-nitrogen-sulfur-nickel networks. CNTs = carbon nanotubes. GCE = Glassy carbon electrode

Reference	Catalyst	Working electrode Support (area)	Loading (mg cm^{-2})	Electrolyte	Overpotential at 10 mA cm^{-2} (mV)	Tafel slope (mV dec^{-1})
Herein	Ni_3S_2 based CNNs: before ADT	Carbon paper (1 cm^2)	0.5	1 M KOH	335	148
	Ni_3S_2 based CNNs: after ADT				194	84
	Pt/Vulcan		0.1		92	49
Int. J. Hydrogen Energy, 2019, 44, 2685-2693.(Oluigbo et al., 2019)	Ni nanoparticles encapsulated in CNTs	GCE (0.07 cm^2)	0.36	1 M KOH	266	102
J. Am. Chem. Soc., 2019, 141, 7537-7543. (Yu et al., 2019)	Ni_2P nanoarray	Ni foam (2.5 × 2.5 cm^2)	-	1 M KOH	37	76
J. Electrochem. Soc., 2019, 166, F168-F173. (Wang et al., 2019)	Ni/Ni_3S_2	Ti mesh (2 cm^2)	-	1 M KOH	441	195
	$NiCu_{0.57}/Ni_3S_2$				239	86
Catal. Sci. Technol., 2016, 6, 1077-1084. (Jiang et al., 2016)	NiS	GCE (0.07 cm^2)	0.283	1 M KOH	474	124
	Ni_3S_2				335	97
	NiS_2				454	128
Angew. Chem. Int. Ed., 2015, 54, 5331-5335. (Yu et al., 2015)	NiS	Ni foam	1	1 M KOH	94	139

Supplementary Table S10| Comparative performance of relevant OER on Ni-based catalysts from literature in alkaline solution. CNNs = carbon-nitrogen-sulfur-nickel networks.

Reference	Catalyst	Working electrode Support (area)	Loading (mg cm^{-2})	Electrolyte	Overpotential at 10 mA cm^{-2} (mV)	Tafel slope (mV dec^{-1})
Herein	Ni_3S_2 based CNNs: before ADT	Carbon paper (1 cm^2)	0.5	1 M KOH	398	91
	Ni_3S_2 based CNNs: after ADT				360	35
	Pt/Vulcan		0.1		435	62
Front. Chem., 2019, 7, Article Number: 523. (Xiao et al., 2019)	Fe-Ni_2P@N,P-CNSs	GCE (0.196 cm^2)	-	0.1 M KOH	390	96
ACS Catal., 2019, 9, 5025-5034. (Suryawanshi et al., 2019)	Ni:FeOOH Nanosheets on 3D N-Doped Graphite Foam	N-Doped Graphite Foam (2 cm^2)	-	1 M KOH	214	36
Advanced Science, 2020, 7, Article Number: 1901833. (Li et al., 2020)	Ru_1Ni_1-nitrogen-doped carbon nanofibers	GCE (0.196 cm^2)	0.612	1 M KOH	290	-
Adv. Funct. Mater., 2016, 26, 4067-4077. (Wang et al., 2016)	Nickel phosphide (Ni-P)	Carbon paper	25.8	1 M KOH	190	-

References

Jiang, N., Tang, Q., Sheng, M., You, B., Jiang, D.-e., and Sun, Y. (2016). Nickel sulfides for electrocatalytic hydrogen evolution under alkaline conditions: a case study of crystalline NiS, NiS2, and Ni3S2 nanoparticles. *Catal. Sci. Technol.* 6(4)**,** 1077-1084. doi: 10.1039/C5CY01111F.

Li, M., Wang, H., Zhu, W., Li, W., Wang, C., and Lu, X. (2020). RuNi Nanoparticles Embedded in N-Doped Carbon Nanofibers as a Robust Bifunctional Catalyst for Efficient Overall Water Splitting. *Advanced Science* 7(2)**,** Article Number: 1901833. doi: 10.1002/advs.201901833.

Oluigbo, C.J., Xie, M., Ullah, N., Yang, S., Zhao, W., Zhang, M., et al. (2019). Novel one-step synthesis of nickel encapsulated carbon nanotubes as efficient electrocatalyst for hydrogen evolution reaction. *Int. J. Hydrogen Energy* 44(5)**,** 2685-2693. doi: https://doi.org/10.1016/j.ijhydene.2018.11.215.

Suryawanshi, M.P., Ghorpade, U.V., Shin, S.W., Suryawanshi, U.P., Jo, E., and Kim, J.H. (2019). Hierarchically Coupled Ni:FeOOH Nanosheets on 3D N-Doped Graphite Foam as Self-Supported Electrocatalysts for Efficient and Durable Water Oxidation. *ACS Catal.* 9**,** 5025-5034. doi: 10.1021/acscatal.9b00492.

Wang, J., Wang, Y., Yao, Z., Xie, T., Deng, Q., Jiang, Z., et al. (2019). Enhanced Hydrogen Evolution Activity of Ni/Ni3S2 Nanosheet Grown on Ti Mesh by Cu Doped Ni. *J. Electrochem. Soc.* 166(2)**,** F168-F173. doi: 10.1149/2.1401902jes.

Wang, X., Li, W., Xiong, D., Petrovykh, D.Y., and Liu, L. (2016). Bifunctional Nickel Phosphide Nanocatalysts Supported on Carbon Fiber Paper for Highly Efficient and Stable Overall Water Splitting. *Adv. Funct. Mater.* 26(23)**,** 4067-4077. doi: 10.1002/adfm.201505509.

Xiao, Y., Deng, S., Li, M., Zhou, Q., Xu, L., Zhang, H., et al. (2019). Immobilization of Fe-Doped Ni2P Particles Within Biomass Agarose-Derived Porous N,P-Carbon Nanosheets for Efficient Bifunctional Oxygen Electrocatalysis. *Front. Chem.* 7(Article Number: 523). doi: 10.3389/fchem.2019.00523.

Yu, X.-R., Liu, F., Wang, Z.-Y., and Chen, Y. (1990). Auger parameters for sulfur-containing compounds using a mixed aluminum-silver excitation source. *Journal of Electron Spectroscopy and Related Phenomena* 50(2)**,** 159-166. doi: https://doi.org/10.1016/0368-2048(90)87059-W.

Yu, X.-Y., Yu, L., Wu, H.B., and Lou, X.W. (2015). Formation of Nickel Sulfide Nanoframes from Metal–Organic Frameworks with Enhanced Pseudocapacitive and Electrocatalytic Properties. *Angew. Chem. Int. Ed.* 54(18)**,** 5331-5335. doi: 10.1002/anie.201500267.

Yu, X., Yu, Z.-Y., Zhang, X.-L., Zheng, Y.-R., Duan, Y., Gao, Q., et al. (2019). “Superaerophobic” Nickel Phosphide Nanoarray Catalyst for Efficient Hydrogen Evolution at Ultrahigh Current Densities. *J. Am. Chem. Soc.* 141(18)**,** 7537-7543. doi: 10.1021/jacs.9b02527.
